# Epitaxial Bi_2_O_2_Se/Bi_2_O_5_Se Thin Films: Revealing Electric‐Field‐Driven Oxidation and Resistive Switching Dynamics for Advanced Memory Devices

**DOI:** 10.1002/advs.75508

**Published:** 2026-05-07

**Authors:** Yen‐Jung Chen, Yong‐Jyun Wang, Zi‐Qin Hong, Chien‐Hua Wang, Yi‐Ning Wang, Jia De Cheng, Chun‐Wei Huang, Ying‐Hao Chu, Wen‐Wei Wu

**Affiliations:** ^1^ Department of Materials Science and Engineering National Yang Ming Chiao Tung University Hsinchu Taiwan; ^2^ Department of Materials Science and Engineering National Tsing Hua University Hsinchu Taiwan; ^3^ Department of Materials Science and Engineering Feng Chia University Taichung Taiwan; ^4^ Advanced Semiconductor Technology Research Center Hsinchu Taiwan

**Keywords:** 2D, atomic‐scale scanning transmission electron microscopy (STEM), bismuth oxyselenide, in situ TEM, resistive random‐access memory (RRAM)

## Abstract

Resistive random‐access memory (RRAM) is a promising technology for nonvolatile applications and neuromorphic computing. 2D bismuth oxyselenide (Bi_2_O_2_Se) exhibits high air stability, carrier mobility, and process compatibility. Moreover, Bi_2_O_2_Se naturally oxidizes into a high‐κ insulating oxide, Bi_2_O_5_Se, making it an ideal candidate for fabricating a dielectric layer in RRAM devices with tunable resistive properties. In this study, RRAM devices based on Bi_2_O_2_Se/Bi_2_O_5_Se bilayer dielectric thin films were epitaxially grown on (001) Nb‐doped SrTiO_3_ substrates. The devices exhibited an exceptional nonvolatile performance, including an endurance of 3 × 10^6^ pulse cycles, retention exceeding 10^4^ s, and stable multilevel resistance states. Additionally, the synaptic‐like behavior was explored by applying pulses to the devices. High‐resolution in situ transmission electron microscopy and aberration‐corrected scanning transmission electron microscopy were used to examine the bilayer structural evolution before and after electric‐field application. The electric‐field‐driven oxidation of Bi_2_O_2_Se was demonstrated, and a new oxygen‐deficient phase was explored during switching. These results established a resistance‐switching mechanism in the Bi_2_O_2_Se/Bi_2_O_5_Se platform for fast and reliable RRAM and neuromorphic operations.

## Graphical abstract

Bi_2_O_2_Se/Bi_2_O_5_Se bilayer heterostructure exhibits reversible structural evolution during device operation, directly revealed by atomic‐scale investigation. Oxygen vacancy redistribution governs filament formation and rupture, enabling stable multilevel resistive switching and synaptic‐like behaviors. This layered architecture provides a pathway toward reliable 2D memory systems.

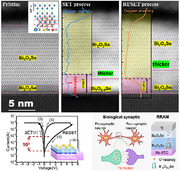

## 1 Introduction

As the demand for electronics and artificial intelligence (AI) technologies has increased, traditional memory has encountered a bottleneck in meeting the computing performance and energy efficiency requirements of next‐generation applications. Resistive random‐access memory (RRAM) has emerged as a promising candidate for neuromorphic engineering, offering nonvolatile operation and the ability to emulate the synaptic functions of the human brain [1, 2, 3, 4, 5]. RRAM has demonstrated various essential synaptic behaviors with a simple two‐terminal structure, including potentiation, depression, and spike‐timing‐dependent plasticity (STDP) [6, 7, 8]. Furthermore, it is widely regarded as a memristor because its resistance can be modulated by voltage sweeps or pulses [9]. The transition from a high‐resistance state (HRS) to a low‐resistance state (LRS) is defined as the SET process, whereas the reverse transition corresponds to the RESET process. These switching behaviors are typically categorized Into several mechanisms: Valence change memory (VCM), which is driven by the migration of oxygen vacancies in transition‐metal oxides [10, 11]; electrochemical metallization (ECM), governed by the formation and rupture of metallic filaments Through cation migration [12, 13]; and topotactic phase‐transition (perovskite–brownmillerite) in which resistance changes arise From structural transformations in the arrangement of oxygen within a crystal lattice while preserving the framework [14, 15].

Developing high‐performance devices with specific functionalities depends on the selection of the switching‐layer materials. In addition to conventional metal oxides and phase‐change materials, 2D layered materials have garnered considerable interest because of their atomic‐scale thicknesses and highly tunable electrical properties, which can be achieved through interface engineering, thickness control, and band‐structure modulation [16, 17, 18]. These unique features have demonstrated great potential in advanced transistor architectures and logic systems and are attractive candidates for memristive devices and artificial synapses [19, 20, 21, 22].

Among the emerging 2D semiconductors, bismuth oxyselenide (Bi_2_O_2_Se) has recently attracted substantial attention owing to its unique layered structure, high carrier mobility, and excellent air stability [23, 24]. These advantages have enabled the broad application of Bi_2_O_2_Se in nanoelectronics and optoelectronics, including in field‐effect transistors [25, 26], photodetectors [27], and sensors [28]. Beyond its superior electronic characteristics, its native oxide, bismuth oxyselenate (Bi_2_O_5_Se), can be generated via thermal oxidation [29], ultraviolet (UV)‐assisted intercalative oxidation [30, 31], or oxygen plasma treatment [32, 33]. The resulting oxide provides a chemically stable interface with a high dielectric constant, making it an ideal gate dielectric. Recent studies have demonstrated α‐Bi_2_SeO_5_ as a high‐κ vdW ferroelectric oxide, which enables high‐performance ferroelectric field‐effect transistors (FeFETs) [34]. This discovery further highlights the potential of the Bi─O─Se material system. Furthermore, Bi_2_O_2_Se has been explored for resistive switching applications, and its intrinsic properties make it a promising candidate for high‐performance memristors [31, 32, 35]. Notably, many of these studies relied on bilayer structures that combined Bi_2_O_2_Se with its oxides. A previous study examined a Bi_2_O_2_Se/Bi_2_SeO_x_ bilayer system [32]; however, it lacked direct microscopic evidence to elucidate the structural and chemical evolution across the stack during switching. Another study demonstrated a β‐Bi_2_SeO_5_/Bi_2_O_2_Se heterostructure memristor via UV‐assisted oxidation [31]; however, the β phase could not be reliably achieved through conventional thermal oxidation, which limited its practical applicability. By contrast, α‐Bi_2_SeO_5_ can be obtained through thermal annealing oxidation; however, its feasibility as an active memristive layer remains unclear. Therefore, the voltage‐driven oxidation dynamics and atomic‐scale switching mechanism in Bi_2_O_2_Se/α‐Bi_2_SeO_5_ bilayers remain insufficiently understood.

In this study, we achieved the heteroepitaxial growth of Bi_2_O_2_Se thin films on Nb‐doped SrTiO_3_ (Nb–STO) substrates by pulsed laser deposition (PLD), followed by controlled annealed oxidation to form a Bi_2_O_2_Se/α‐Bi_2_O_5_Se heterostructure. This method is illustrated in Figure S1a. This Au/Ti/Bi_2_O_2_Se/α‐Bi_2_O_5_Se/Nb–STO device exhibited robust resistive switching with a high on/off ratio and excellent endurance over 10^7^ cycles. Advanced in situ transmission electron microscopy (TEM) and aberration‐corrected scanning transmission electron microscopy (STEM) demonstrated the atomic‐scale structural evolution of the bilayer system and its interface, thereby clarifying the resistive switching mechanism. X‐ray photoelectron spectroscopy (XPS) and electron energy loss spectroscopy (EELS) were employed to verify the chemical state changes associated with different resistance states. These findings demonstrated the potential of integrating 2D Bi_2_O_2_Se/α‐Bi_2_O_5_Se heterostructures into RRAM technology, providing a viable path to next‐generation memories while emulating fundamental synaptic‐Like behaviors, thus positioning them as promising candidates for neuromorphic computing in AI hardware.

## 2 Results

**FIGURE 1:**
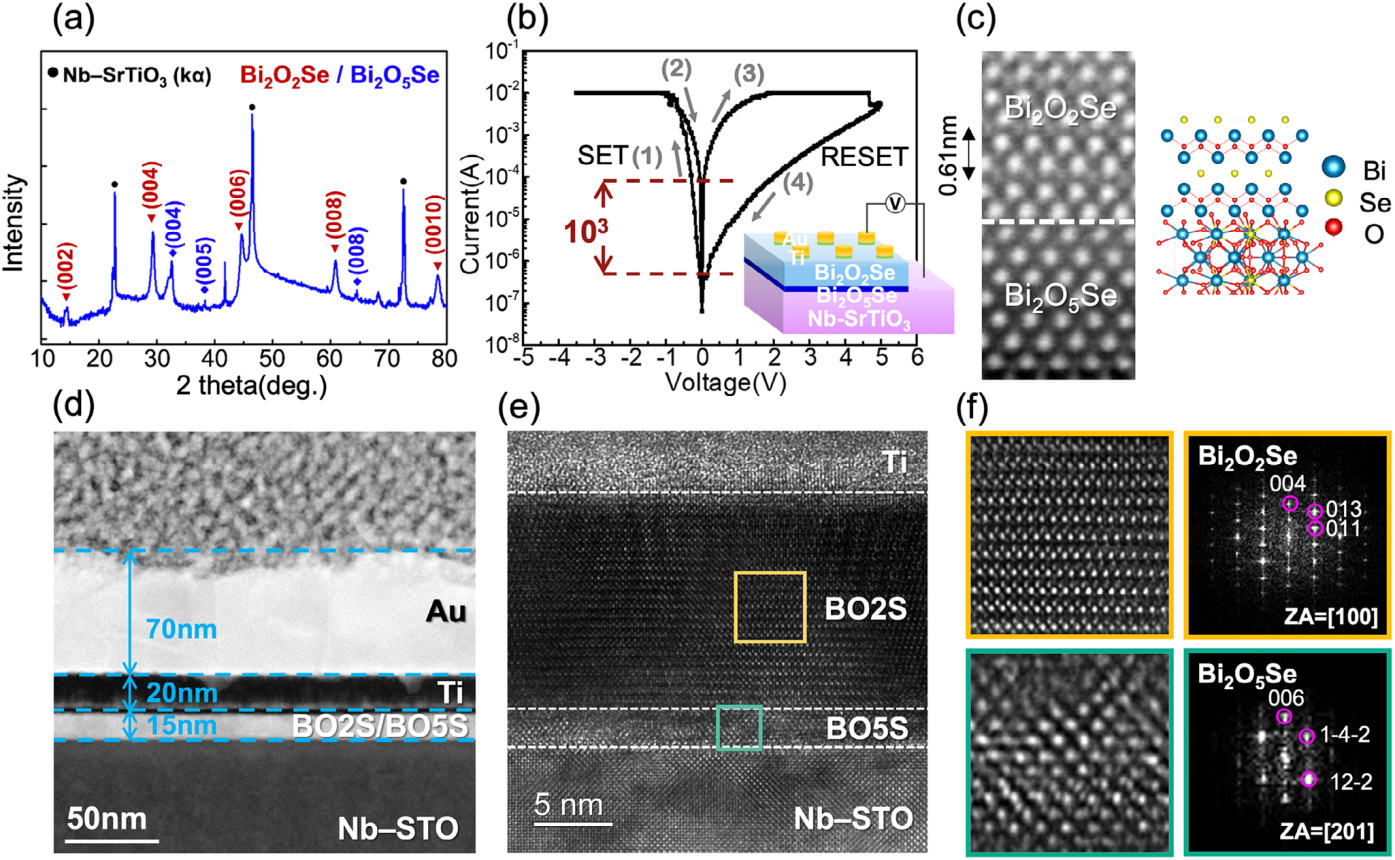
Basic characterization of the fabricated devices. (a) XRD pattern of Bi_2_O_2_Se/Bi_2_O_5_Se/Nb‐STO device. (b) *I–V* characteristic curves and the schematic structure of the devices. The continuous DC voltage sweep was performed in the sequence of (1) 0 → −3.5 V; (2) −3.5 → 0 V; (3) 0 → +5 V; and (4) +5 → 0 V, as indicated by the numbered arrows. The device exhibits non‐volatile bipolar resistive switching, with the SET process occurring under negative bias applied to the top electrode and the RESET process observed under positive bias. (c) Atomic‐resolution STEM image and the atomic model of the bilayer system. The blue, yellow, and red spheres represent Bi, Se, and O, respectively. (d) Low‐magnification STEM image. (e) High‐resolution TEM image. (f) Yellow and green regions indicated in Figure 1e correspond to Bi_2_O_2_Se(top) and Bi_2_O_5_Se (bottom) layers, respectively, as identified by the diffraction patterns.

Figure 1a presents the X‐ray diffraction (XRD) pattern of the fabricated device. The (002) series signals of Bi_2_O_2_Se were observed Along With the (001) series peaks of the Nb─STO substrate. Furthermore, a significant Bi_2_O_5_Se (004) peak appeared near 32°, confirming the successful growth of a well‐defined Bi_2_O_2_Se/Bi_2_O_5_Se stack on Nb─STO. Optical microscopy (OM) and Raman spectroscopy were employed to distinguish the bilayer from a single Bi_2_O_5_Se film. In the OM images (Figure S2a,b), a clear color contrast is observed; the sample with the top Bi_2_O_2_Se layer appears lighter, whereas the single‐layer Bi_2_O_5_Se sample exhibits a darker contrast. This contrast originated from the distinct optical properties of the two oxide layers, which allowed the underlying Nb–STO substrate to be more visible in the Bi_2_O_5_Se device. The Raman spectra (Figure S2c,d) further support this distinction, with the Bi_2_O_2_Se/Bi_2_O_5_Se film showing a characteristic peak at approximately 159 cm^−1^ and the pure Bi_2_O_5_Se film exhibiting a peak at approximately 250 cm^−1^ [36, 37]. For comparison, the XRD pattern of a single Bi_2_O_5_Se layer is shown in Figure S3a. These observations confirmed that after annealing and subsequent deposition, the additional Bi_2_O_2_Se layer was successfully stacked on the Bi_2_O_5_Se film, thereby forming the intended bilayer structure. Electrical characterization was performed by grounding the Nb─STO substrate and applying a voltage sweep to the Au/Ti top electrode. The current–voltage (*I–V*) characteristics were investigated in a sequence of 0 → ‐3.5 → 0 → 5 → 0 V, which is shown in Figure 1b. The inset shows a schematic of the device structure. The device initially exhibited the HRS, and the current gradually increased with increasing negative voltage. A transition switch to the LRS occurred at V_set_ ≈ −1.5 V, where the current reached the compliance limit of 10 mA. The device remained in the LRS until a positive bias reset it back to the HRS at V_reset_ ≈ 5 V. The on/off ratio exceeded three orders of magnitude, providing a clear and distinguishable resistance window. Figure 1c shows an atomic‐resolution STEM image of the Bi_2_O_2_Se/Bi_2_O_5_Se heterostructure and a structural model of the bilayer system. The upper layer, Bi_2_O_2_Se, exhibited a tetragonal crystal structure (space group *I*4/*mmm*, *a*  =  *b*  =  3.88 Å, *c*  =  12.16 Å). With a characteristic thickness of approximately 6.1 Å confirms its van der Waals stacking nature. By contrast, the underlying layer, Bi_2_O_5_Se, crystallized in an orthorhombic phase (space group Abm2, a = 11.42 Å, b = 16.24 Å, c = 5.49 Å) [29]. The STEM image of a single Bi_2_O_5_Se layer is shown for comparison in Figure S3c. The low‐magnification cross‐sectional STEM image in Figure 1d shows the complete device stack, including the Au/Ti electrodes (70 nm/20 nm) and the Bi_2_O_2_Se/Bi_2_O_5_Se bilayer, with a total thickness of approximately 15 nm. High‐resolution TEM (Figure 1e) reveals a single‐crystalline structure and an atomically sharp interface; these observations are further supported by a large‐area aberration‐corrected STEM image (Figure S1b), which confirms the high‐quality epitaxial growth of the Bi_2_O_2_Se/Bi_2_O_5_Se thin films via PLD. Figure 1f shows enlarged regions and corresponding fast Fourier transform diffraction patterns (FFT–DPs) of the area marked in Figure 1e. The upper layer, indexed along the [100] zone axis, corresponds to Bi_2_O_2_Se, whereas the underlying layer, indexed along the [201] zone axis, is assigned to Bi_2_O_5_Se. These observations verified the structural integrity of the bilayer stack in our RRAM device

**FIGURE 2:**
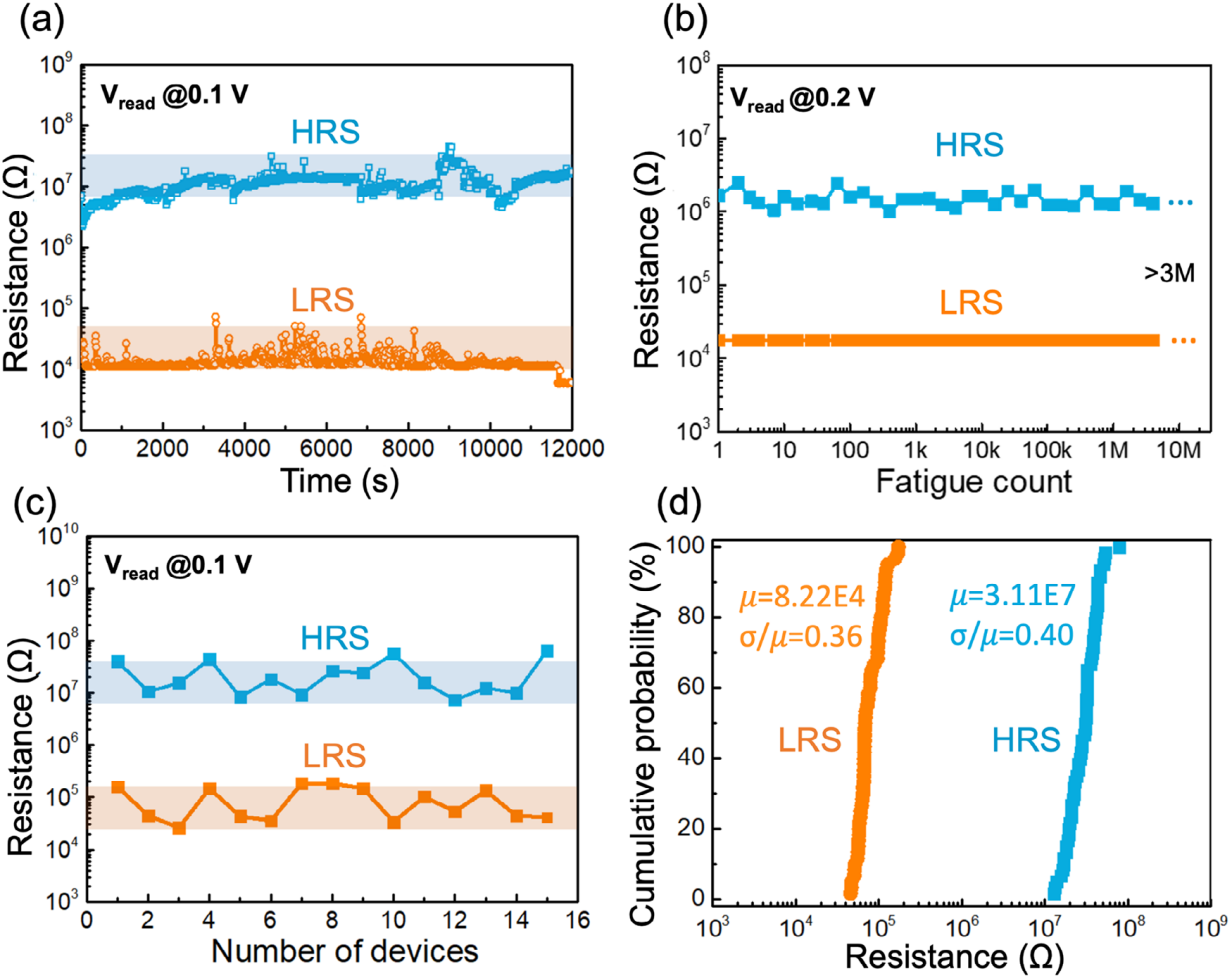
Electrical performance of the Bi_2_O_2_Se/Bi_2_O_5_Se/Nb–STO RRAM devices. (a) Retention test results showing that both HRS and LRS remained stable for over 12000 s under a reading voltage of 0.1 V. (b) Endurance test demonstrating reliable switching over 3 million cycles with the pulse amplitude and width set to −3.5 V/200 µs for writing operation and 5 V/100 µs for erasing operation, respectively, with a reading voltage of 0.2 V. (c) On/off ratios measured at a reading voltage of 0.1 V across 15 devices. (d) Cumulative probability distribution of the resistance values. The coefficient of variation of the HRS is 0.40, whereas that of the LRS is 0.36.

The electrical characteristic results of Au/Ti/Bi_2_O_2_Se/α‐Bi_2_O_5_Se/Nb–STO devices are presented in Figure 2. Figure 2a shows the retention measurement results, in which the on/off ratio is maintained for more than 12 000 s. A slight upward drift is observed in the HRS, whereas the LRS remains nearly constant. The endurance was evaluated using continuous pulse measurements (Figure 2b), demonstrating stable switching over more than 3 × 10^6^ cycles. The device‐to‐device variation (Figure 2c) shows consistent on/off ratios across the 15 devices, confirming the consistency and reproducibility of the results. As shown in Figure 2d, the cumulative probability distribution of the HRS and LRS resistance values exhibits low variability. Figure S4a shows the *I–V* characteristics of 420 successive cycles with good uniformity. To investigate the multilevel data storage capability, we controlled the current compliance limit (I_cc_) to yield eight clearly distinguishable *I–V* curves (Figure S4b). Additionally, the ability to maintain a multilevel endurance status was assessed. I_cc_ varied from 1 to 15 mA during the SET process, and different LRS states were obtained (Figure S4c). Applying a higher current limit reinforced the conductive filaments, resulting in lower resistance. The resistances in each LRS exhibited stable retention for more than 1000 s, indicating that the device had good data‐retention performance. Furthermore, we investigated the use of different reset voltages ranging from 4 to 5.5 V (Figure S4d). Higher reset voltages resulted in higher resistance states. The multilevel resistance state for each HRS remained unchanged for 600 s, confirming the robust multilevel retention behavior of this system.

To explore the resistance‐switching mechanism of the devices, the *I–V* characteristics were analyzed using the ln(I)–ln(V) plots under both bias polarities (Figure S5a,b). During the SET sweep, the charge transport in the HRS was dominated by the space‐charge‐limited current (SCLC) process [38, 39]. The ln(I)–ln(V) slope evolves as 2.2→4.1→2.12 as the negative voltage increases. In the low‐voltage region, the slope near 2 indicates a trap‐free SCLC. The charge could be injected from the electrode into the bulk, and it had difficulty moving further through the material. In the intermediate‐voltage regime, the slope increases to 4.1, indicating that the trap‐controlled SCLC is approaching the trap‐filled limit (TFL). Here, V_TFL_ = 0.86 V, which is defined as the threshold voltage required to fill the traps. Beyond the TFL, the traps become filled, the current rapidly returns to the trap‐free SCLC state, and the slope decreases to approximately 2. Upon switching to the LRS, the transport became Ohmic‐like, which was consistent with the formation of a continuous conductive path. During the RESET process under a positive bias, Ohmic conduction dominated at a low bias, yielding a fitted slope of approximately 1.19 with R^2^ ≈ 0.993. By contrast, Schottky emission governed the high‐voltage region, as evidenced by the linear ln(I)–V^1/2^ relationship with a high fitting quality (R^2^ ≈ 0.999). To further investigate the temperature‐dependent conduction behavior, the HRS and LRS were measured at various temperatures (298, 323, 333, and 358 K; Figure S6a). The on/off ratio at each temperature remained constant for more than 100 s, indicating the excellent thermal stability and retention characteristics of each state. The temperature‐dependent resistance of the HRS was analyzed using the Arrhenius equation to elucidate the conduction mechanism (Figure S6b). The plot of ln(*R*/*R*_0_) versus 1/*T* exhibited a linear dependence, which is as per the following Arrhenius equation [40]:

$$R\left(T\right)=R_{0}\exp\left(\frac{E_{a}}{k_{B}T}\right)$$

where *k*_B_ is the Boltzmann constant. The fitted slope yielded an activation energy of E_a_ ≈ 0.27 eV, indicating the energy barrier that charge carriers must overcome during transport. This behavior suggested carrier transport through localized trap states, which may be related to oxygen vacancy defects. By contrast, the temperature‐dependent resistance of the LRS is shown in Figure S6c. Here, R/R_0_ weakly decreases with increasing temperature but does not follow a linear Arrhenius behavior, indicating that charge transport was dominated by a defect‐assisted conduction pathway rather than thermally activated hopping [41].

**FIGURE 3:**
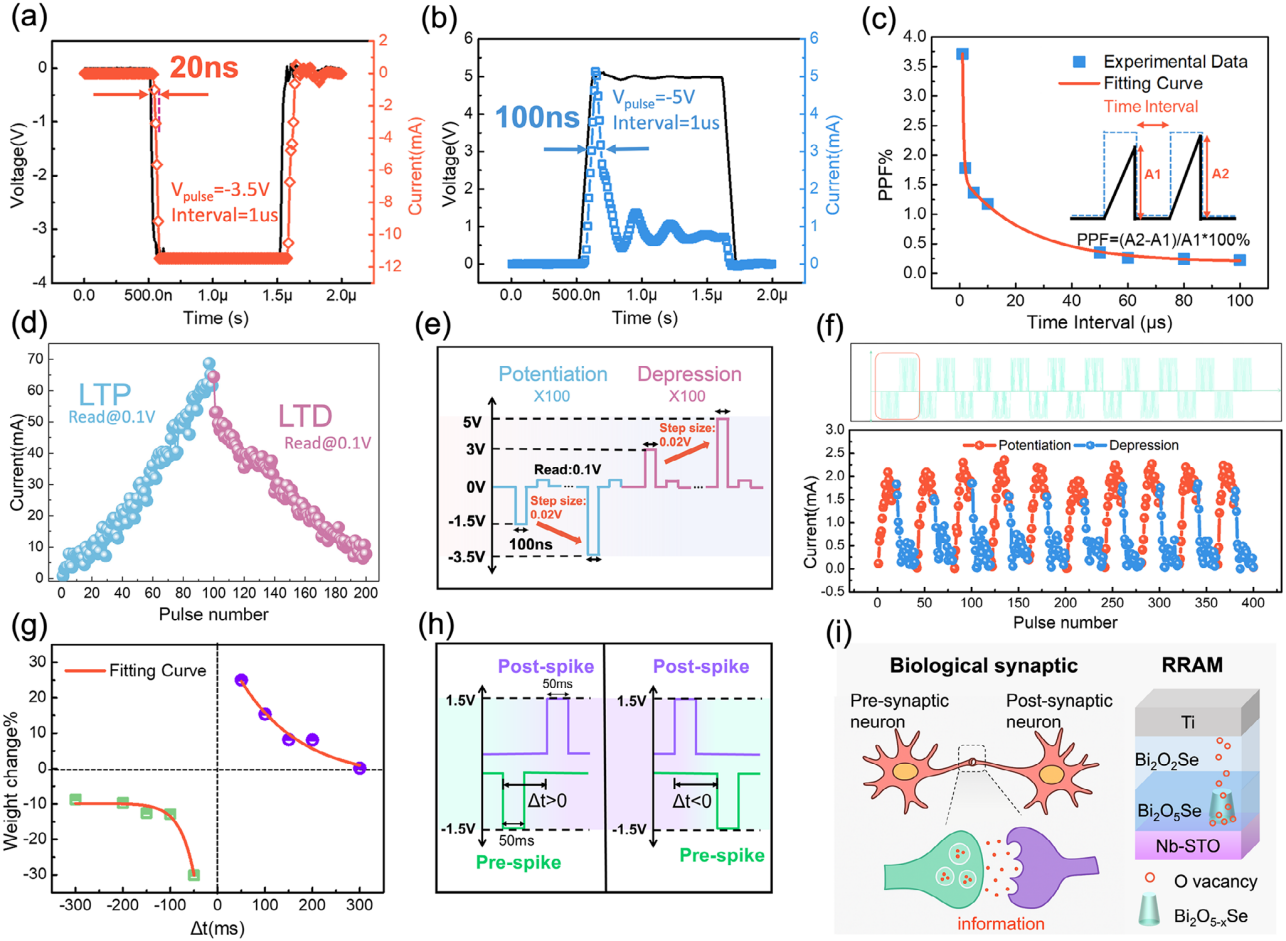
Synaptic behaviors of Bi_2_O_2_Se/Bi_2_O_5_Se devices for neuromorphic computing. (a, b) Switching‐speed measurement results of the RRAM device under pulses with an amplitude/width of (a) −3.5 V/1 µs, (b) 5 V/1 µs. (c) Paired‐pulse facilitation (PPF) index as a function of the pulse interval (Δt). The fitting curve matches the experimental data. (d) Demonstration of the LTP and LTD characteristics with the gradual increase in pulse amplitude to reach near‐linear potentiation and depression. (e) Pulse diagram used for 200 potentiation and depression. (f) Ten cycles of successive operations under 400 pulses. (g) Spike‐timing‐dependent plasticity (STDP) characterization, illustrating the change in synaptic weight as a function of the time difference (Δt) between pre‐ and post‐synaptic spikes. (h) Pulse diagram used for STDP characterization. (i) Schematic comparing the biological synapse and RRAM‐based devices.

To realize neuromorphic computing systems, we emulated synapse‐like dynamics in our RRAM devices. Pulse measurements for paired‐pulse facilitation (PPF), long‐term potentiation and long‐term depression (LTP/LTD), and STDP were performed to verify these functions. Fast‐switching dynamics were observed. The response times were approximately 20 and 100 ns for the SET and RESET processes, respectively (Figure 3a,b). Next, we mimicked the PPF phenomenon in the biosynapses. Figure 3c shows PPF as a function of the time interval between the two pulses. The PPF value is defined as ((A2 – A1)/A1) × 100%, where A1 and A2 are the currents of the first and second pulse inputs, respectively [42]. The first short pulse could not induce sufficient mobile species to form a complete conductive path, whereas the subsequent pulse arriving before relaxation enhanced carrier accumulation, leading to an increase in conductance similar to that promoted by biological PPF. Establishing several learning dynamics for LTP and LTD was essential for emulating synaptic plasticity and enabling the gradual and reversible conductance modulation required for neuromorphic computing [43, 44, 45]. Figure S7b illustrates the potentiation and depression cycles using 40 pulses, applied at −1.8 and 2.5 V, respectively, with a constant width of 100 ns and 0.1 V read pulses (Figure S7c). Our RRAM devices exhibited nonlinear conductance modulation owing to vacancy migration and filament saturation. However, gradually increasing the pulse amplitude or width enabled near‐linear potentiation and depression [46, 47]. This approach led to a gradual enhancement in conductance due to stronger oxygen vacancy migration and filament growth, whereas reverse pulses induced filament rupture and depression. 200 potentiation and depression pulses were performed using a 0.1 V read voltage in Figure 3d. The pulse amplitude was increased from −1.5 to −3.5 V for potentiation and from 3.0 to 5.0 V for depression, both with a step size of 0.02 V and a pulse width of 100 ns (Figure 3e). This highly linear and uniform conductance update is essential for minimizing training errors and ensuring accurate synaptic weight modulation in practical neuromorphic systems. Ten potentiation and depression cycles were further conducted (Figure 3f), demonstrating stable pulse measurements without degradation. The effects of the pulse amplitude and width on the performance of LTP and LTD were also studied. Different pulse amplitudes (−1.8, −2.5, and −3.5 V) were applied at a fixed pulse width of 1 µs, and 40 continuous pulses were used to simulate the potentiation and depression behaviors (Figure S7e). Similarly, different pulse widths (0.1, 1, and 10 µs) were applied at a fixed pulse amplitude of −3.5 V (Figure S7f). In both cases, the conductance during potentiation increased with the pulse amplitude and width. The weights of the synapses could be modulated by changing the resistance states in RRAM devices, following the rule of STDP [6, 45]. In biological synapses, when a pre‐synaptic neuron fires before a post‐synaptic neuron, the synaptic weight increases (w>0), leading to LTP. Conversely, if the post‐synaptic neuron fires prior to the pre‐synaptic neuron, the synaptic weight decreases (w<0), resulting in LTD. In our RRAM devices, the synaptic weight could be tuned by precisely controlling the relative timing between the pre‐ and post‐synaptic spikes. Figure 3g shows the experimental results of the relationship between the time difference (Δt) and corresponding synaptic weight change (Δw). A smaller time interval (|∆t|) between the two spikes produced a larger conductance change, consistent with biological synapses. Pre‐ and post‐synaptic voltage pulses (−1.5 V, 50 ms) were applied to the top and bottom electrodes, respectively (Figure 3h). The change in synaptic weight was quantified as (*G*_1_ – *G*_0_)/*G*_0_ ×100%, where *G*_0_ and *G*_1_ are the conductance after pre‐ and post‐synaptic spikes, respectively. The time difference between the spikes was defined as Δt = t_post—_t_pre._ Figure 3i shows a comparison between the biological synapse and RRAM‐based devices. The top and bottom electrodes are the pre‐ and post‐synaptic neurons in the human brain, respectively, whereas the conductive filament serves as an artificial synaptic channel that transmits information between the two terminals. A series of measurements on the Au/Ti/Bi_2_O_2_Se/α‐Bi_2_O_5_Se/Nb–STO devices demonstrated that our RRAM devices could emulate fundamental synaptic‐like behaviors, making them promising candidates for neuromorphic computing in AI hardware.

**FIGURE 4:**
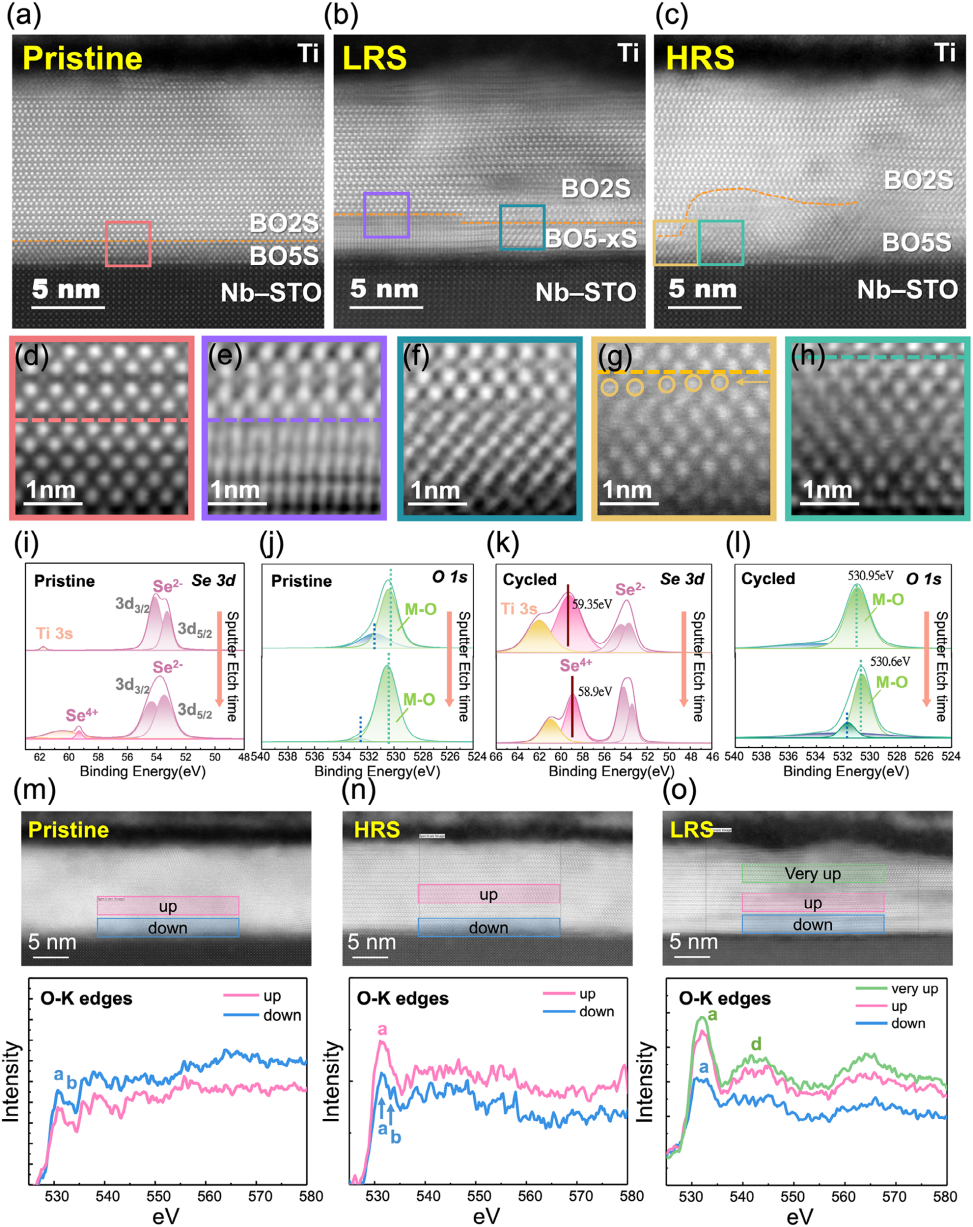
Atomic‐scale STEM images and XPS/EELS characterization. STEM images in the (a) pristine, (b) cycled‐LRS, and (c) cycled‐HRS states. The dashed lines represent the interface between the upper and bottom layers. (d–h) Enlarged STEM images corresponding to regions indicated in Figure 4a–c. XPS spectra of (i) Se 3d, and (j) O 1s for the pristine Bi_2_O_2_Se/Bi_2_O_5_Se heterostructure. XPS spectra of (k) Se 3d, and (l) O 1s for the cycled Bi_2_O_2_Se/Bi_2_O_5_Se heterostructure. EELS spectra at the O–K‐edge corresponding to the (m) pristine; (n) cycled‐HRS; and (o) cycled‐LRS states.

We further conducted atomic‐scale STEM imaging to investigate the resistive switching behavior of the RRAM devices (Figure 4). Figure 4a shows the pristine state with a clear interface between the top Bi2O2Se (BO2S) and bottom Bi2O5Se (BO5S) layers. The magnified image, highlighted within the red square, shows an atomically sharp interface with a uniform lattice contrast (Figure 4d). After the electrical operation, STEM analysis was performed to examine the LRS and HRS characteristics of the devices. The LRS was obtained after applying a negative bias, as shown in Figure 4b. Slight undulations appeared near the interface, and the bottom BO5S layer was partially reduced to an oxygen‐less Bi2O5‐xSe (BO5‐xS) layer, indicating the formation of conductive regions. This oxygen reduction was driven by the electric field, and under a negative bias, O2^−^ ions migrated downward toward the Nb─STO substrate, leading to an increased vacancy concentration within the heterostructure. As a result, these locally formed vacancies within the BO2S layer could connect to the oxygen‐deficient region in the underlying BO5‐xS layer, creating a continuous pathway for carrier transport and yielding the LRS. After multiple voltage sweeps, the device was placed under the HRS (Figure 4c). The bottom oxide exhibited a BO5S structure. Additionally, the bottom layer gradually thickened owing to the conversion of the partial upper BO2S layer into BO5S, indicating oxygen incorporation into the upper layer under a positive bias. Figure S8 shows the energy‐dispersive X‐ray spectroscopy (EDS) elemental maps of the initial and cycled devices. The elemental distributions remained uniform without detectable interdiffusion or significant compositional loss, confirming that the switching mechanism was independent of electrode diffusion. To further observe the conductive regions, a wide‐field TEM image of the cycled device is presented in Figure S9a. The enlarged regions A and B show distinct structural features in the bottom layer after electrical cycling. The coexistence of Bi2O5Se and Bi2O5‐xSe confirmed that switching occurs through laterally localized transformation rather than a global breakdown of the entire film. Figure S9b shows the vertical structural distortion across the BO2S layer, implying the presence of a continuous defect‐mediated conduction path for electron migration. To better understand oxidation within the BO2S layer under a positive bias, the structural evolution at the BO2S/BO5S interface was observed, as shown in Figure S10a. The line profiles exhibit an enlarged van der Waals spacing, indicating lattice expansion associated with oxygen insertion and the subsequent rearrangement of the Bi─O coordination framework. Furthermore, the EDS results in Figure S10b indicate a relative decrease in the oxygen concentration within both layers at the LRS, suggesting the formation of oxygen‐deficient channels.

High‐resolution X‐ray photoelectron spectroscopy (HRXPS) was used to elucidate the resistive switching mechanism. The XPS depth profile shows the distribution of elements before and after electrical treatment (Figure S11a,b), and the Bi 4f, O 1s, Se 3d, and Ti 2p spectra at different sputtering depths (1–10) are displayed in Figure S11c,d, These measurements demonstrate a clear compositional and chemical‐state gradient across the Bi_2_O_2_Se/Bi_2_O_5_Se stack, which served as the basis for the following detailed analysis. The Se 3d and O 1s spectra of the pristine device are shown in Figure 4i,j. In the Se 3d spectra, the peaks at 53.3 and 54.1 eV correspond to the Se 3d_5/2_ and Se 3d_3/2_ components of Se^2−^ in the BO2S layer, respectively. As the sputtering depth increases, these peaks shift slightly to 53.5 and 54.3 eV, accompanied by a new feature at 59.3 eV, which is attributed to Se^4+^ for Se─O bonding [30] and confirms the formation of the oxidized BO5S layer. The slight upward shift of the Se^2−^ peaks suggests an increased oxidation degree in the bottom layer. In the O 1s spectra, the peaks at 530.4 and 531.5 eV in the upper layer correspond to the Bi─O lattice oxygen and weakly coordinated oxygen [48], respectively. In the deeper region, the peaks shift to 530.5 eV, reflecting a more highly coordinated oxygen environment consistent with the BO5S layer. The Se 3d, O 1s, and Bi 4f spectra of a single Bi_2_O_5_Se layer are shown in Figure S3d for comparison. A distinct Se^4+^ peak at 58.6 eV associated with Se–O bonding is displayed in the Bi_2_O_5_Se layer. After electric treatments (Figure 4k,l), the Se^4+^ peak near 59.3 eV is more significant in the upper region, suggesting the formation of Se─O bonds in the BO2S layer. The bottom region has retained a dominant Se^4+^ component, while the Se^4+^ peak has shifted to a lower binding energy (approximately 58.8 eV), reflecting a partial reduction in the Se–O environment. Similarly, in the corresponding O 1s spectra, the main lattice oxygen peak has increased to 530.9 eV, reflecting a strengthened bonding environment. An additional component at approximately 532.1 eV suggests weakly coordinated oxygen resulting from locally distorted bonding configurations, suggesting structural disorder after electrical cycling. The Bi 4f XPS spectra (Figure S12) exhibit a clear spin‐orbit doublet corresponding to the Bi^3+^ peak located at 158.4 (Bi 4f_5/2_) and 163.7 eV (Bi 4f_7/2_) in the pristine device. In the deeper region, the Bi 4f doublet has shifted slightly toward higher binding energies, consistent with higher oxygen coordination in the BO5S layer. After electrical cycling, the Bi 4f spectra in the upper BO2S region have remained unchanged, indicating that the local Bi chemical environment was preserved. By contrast, the Bi 4f doublet in the bottom region has shifted slightly toward a higher binding energy. This shift suggested enhanced oxygen coordination and local structural relaxation within the Bi─O─Se framework after cycling.

To clarify the presence of oxygen‐deficient environments, EELS was used to examine the O─K edge structures across the Bi_2_O_2_Se/Bi_2_O_5_Se stack in the pristine, cycled‐HRS, and cycled‐LRS states (Figure 4m–o). In the pristine state, both the upper Bi_2_O_2_Se (“up”) and bottom Bi_2_O_5_Se (“down”) regions exhibit a doublet at approximately 531 (feature “a”) and approximately 533 eV (feature “b”). In the cycled‐HRS state, the up (pink) region exhibits a single broadened feature at the “a” peak position, which contributed to an oxygen‐deficient bonding state. These features are associated with locally reduced oxygen environments that remain after switching [49]. By contrast, the down (blue) region retains a clear a–b doublet, indicating a more oxidized and better‐coordinated lattice‐oxygen environment than the upper region. In the cycled‐LRS state, spectra were acquired from three vertical positions (very‐up, up, and down). All display a single broadened band at the “a” peak position, indicating strong oxygen reduction throughout the stack. The “very‐up” region exhibits an additional high‐energy shoulder (“d” peak), reflecting strongly distorted interfacial oxygen coordination [50]. The intermediate “up” region displays mixed characteristics of both reduced and distorted oxygen states. These vertically stacked regions establish a continuous conduction pathway, enabling the LRS. These EELS measurements confirm that the oxygen‐deficient regions establish a continuous vertical conduction pathway to enable the LRS, but it remains localized in the lateral dimension.

The STEM–EELS line‐scan analyses further demonstrated the interfacial evolution of the device under different resistance states (Figure S13). The colored overlays in the STEM images indicate the Bi_2_O_2_Se layer (yellow region) and the Bi_2_O_5_Se layer (pink region). The green rectangle highlights the region of the EELS line‐scan measurements. Figure S13d shows the atomic percentages of O, Se, and Ti in the pristine sample. The oxygen‐rich bottom layer, which lacks a detectable Ti contribution, confirms that the signal originated from the oxidized BO5S film rather than from the substrate. After electrical cycling, the cycled‐HRS results (Figure S13e) show that the oxygen‐rich interfacial layer increases in thickness from 1.94 to 2.46 nm, indicating oxygen incorporation and interfacial growth. Under the cycled‐LRS condition (Figure S13f), the bottom region is still thicker than the pristine state (≈2.98 nm) but displays a significant decrease in the O signal within BO5‐xS (annotated as “oxygen deficiency”), consistent with the formation of oxygen‐deficient states. These results supported an electric‐field‐driven switching mechanism. During the SET process, oxygen is depleted, generating a conductive channel. During the RESET process, oxygen migrated upward and was first reinserted into the vacancy‐rich BO5‐xS layer, while additional oxygen continued to diffuse into the upper BO2S region, partially converting it into BO5S and thereby rupturing the conductive channel.

**FIGURE 5:**
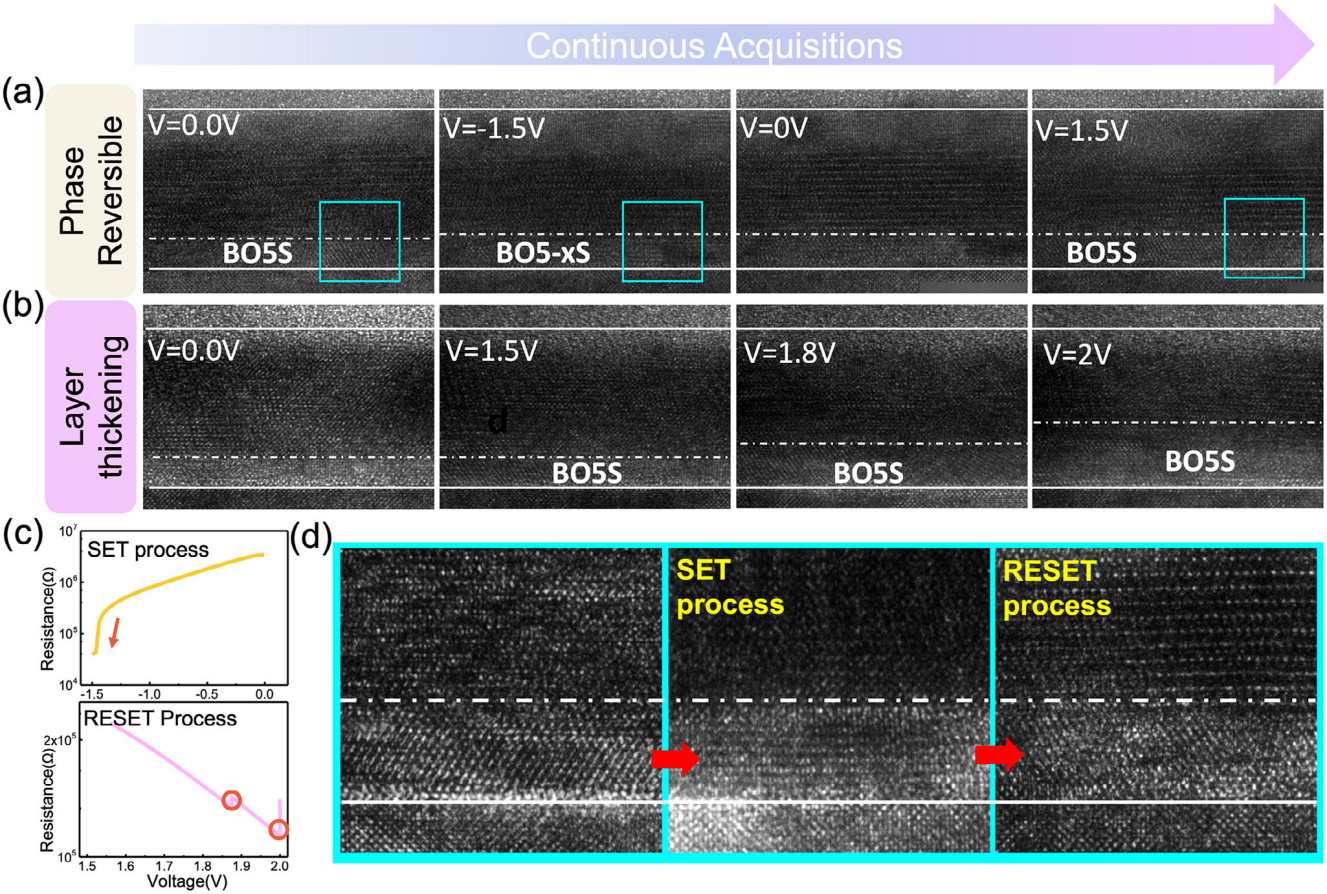
Dynamic in situ TEM observations of the Bi_2_O_2_Se/Bi_2_O_5_Se heterostructure under an electrical bias. (a) A series of in situ TEM images demonstrates a phase change from Bi_2_O_5_Se to Bi_2_O_5‐x_Se under a negative bias, while the Bi_2_O_5‐x_Se structure reverses back to Bi_2_O_5_Se under a positive bias. (b) A series of TEM images demonstrating a bottom‐to‐top transformation of Bi_2_O_2_Se into Bi_2_O_5_Se under a high positive voltage. (c) *I–V* curves obtained during the in situ biasing experiment. (d) Enlarged images obtained from Figure 5a show the structural evolution in the SET and RESET processes.

We demonstrated the dynamics of resistive switching via in situ TEM techniques. Figure 5 shows a time series of in situ TEM images to illustrate the structural transformations that occur under an electrical bias. Figure 5a shows continuous voltage sweeps from 0 to −1.5 V with a 0.1 V/s step to capture the SET process in the Bi_2_O_2_Se/Bi_2_O_5_Se heterostructure. The pristine device exhibited a BO5S structure in the bottom layer. Upon reaching −1.5 V, an abrupt phase transformation occurred, and the bottom oxide was converted to oxygen‐deficient BO5‐xS, marking the transition to the LRS (from 0 to −1.5 V; Movie S1). When the voltage sweep was reversed from −1.5 to 1.5 V, the device initially preserved the oxygen‐deficient phase, indicating the nonvolatile retention of the LRS below the RESET threshold. Once the bias reached 1.5 V, the bottom layer underwent reoxidation to BO5S, thereby rupturing the conductive pathway and restoring the HRS (from 0 to 1.5 V; Movie S2). To minimize beam influence during the switching process, we performed electrical measurements with the electron beam blanked (turned off) while maintaining the sample at the same position. The beam was unblanked briefly after the transition. To further elucidate the interaction within the bilayer heterostructure, the devices were maintained at HRS, and a larger positive bias was applied (1.5–2.0 V; Figure 5b; Movie S3). Under these conditions, the BO5S layer gradually expanded as additional oxygen migrated into the BO2S layer, resulting in bottom‐to‐top oxidation. Oxygen insertion increased the Bi coordination number and directed the reconstructive transition toward the oxygen‐rich Bi_2_O_5_Se phase. The weakly bonded interlayer structure of the BO2S lattice suggested a structurally permissive framework that could allow vertical oxygen incorporation under an electrical bias [51, 52, 53]. However, the conversion did not extend across the entire BO2S thickness, demonstrating that the oxidation of Bi_2_O_2_Se was self‐limited. These structural observations were consistent with the electrical characteristics. First, repeated switching cycles led to a gradual increase in the HRS, indicating the cumulative growth of the insulating region. Second, retention measurements demonstrated a time‐dependent increase in the HRS, which eventually saturated, suggesting that the oxidized phase could reach a stable thickness. Third, the HRS increased monotonically with the applied RESET voltage, indicating that a stronger positive bias drove deeper oxygen insertion and promoted a more extensive conversion from Bi_2_O_2_Se to Bi_2_O_5_Se. Figure S3b shows the *I–V* curve of a single‐layer Bi_2_O_5_Se RRAM device, indicating that it had a higher operating voltage and lower on/off ratio than the bilayer stack. This study provided the first direct evidence via in situ TEM analysis that an applied electric field can vertically oxidize Bi_2_O_2_Se to Bi_2_O_5_Se during device operation. Such a self‐limiting mechanism preserved the van der Waals stacking nature in the Bi_2_O_2_Se framework, which offered a more stable and reversible operation. During the real‐time structural evolution, the device resistance was monitored simultaneously, as shown in Figure 5c. A significant decrease in the resistance occurred during the SET process, whereas during the RESET process, the resistance increased at elevated RESET voltages. Figure 5d presents enlarged views of the stepwise structural transformations. These dynamic in situ observations, consistent with ex situ TEM characterizations, validated a reversible, electric‐field‐driven switching mechanism. Importantly, as the in situ TEM experiments were conducted under a high‐vacuum environment, the possibility of atmospheric oxygen participating in the oxidation process can be ruled out, ensuring that the observed structural evolution is an intrinsic property of the Bi_2_O_2_Se/Bi_2_O_5_Se bilayer.

## 3 Discussions

**FIGURE 6:**
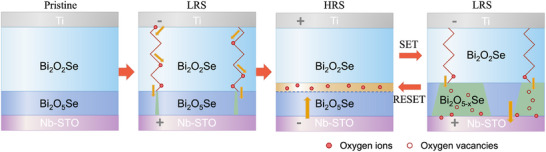
Schematic of the resistive switching process in the Bi_2_O_2_Se/Bi_2_O_5_Se device. The SET and RESET processes illustrate the structural evolution at various stages. Red dots represent oxygen atoms, and yellow arrows indicate that oxygen migrates from the bottom to the top electrode under a positive bias, promoting the oxidation of the Bi_2_O_2_Se layer and yielding a progressive thickening of the Bi_2_O_5_Se layer. The green region represents the oxygen‐deficient Bi_2_O_5‐x_Se region.

According to the electrical measurements and atomic‐scale analysis, we established a resistive switching mechanism for the Bi_2_O_2_Se/Bi_2_O_5_Se heterostructure, as illustrated in Figure 6. When a negative bias was applied to the top electrode, the bottom Bi_2_O_5_Se layer was reduced to oxygen‐deficient (Bi_2_O_5‐x_Se), and additional oxygen depletion also occurred in the upper Bi_2_O_2_Se region. The resulting oxygen‐vacancy‐rich region provided a vertical continuous pathway for electron transport, forming a conductive filament that defined the LRS. Upon applying a positive bias, the oxygen‐deficient Bi_2_O_5‐x_Se region captured oxygen and reverted to the stoichiometric Bi_2_O_5_Se phase. Meanwhile, the intensified electric field drove excess oxygen further upward into the Bi_2_O_2_Se layer, promoting its oxidation and producing a bottom‐to‐top thickening of the Bi_2_O_5_Se region. After repeated SET/RESET cycles, oxidation occurred near the stacked interface, whereas the upper part of the Bi_2_O_2_Se layer remained intact. This self‐limited oxidation effectively ruptured the conductive filament, yielding a tunable HRS. In this bilayer architecture, Bi_2_O_5_Se acted as the primary switching layer, whereas the Bi_2_O_2_Se layer did not serve as the primary switching medium during the resistive switching process. Instead, it provided a stable conduction path that assisted in carrier transport and vacancy redistribution. This stabilizing role homogenized the electric field and mitigated current overshoot during repeated SET/RESET operations, leading to improved endurance and enhanced cycle‐to‐cycle uniformity. Consequently, the Bi_2_O_2_Se/Bi_2_O_5_Se bilayer enabled a lower voltage and more reliable switching than single‐layer Bi_2_O_5_Se devices, confirming the advantages of the bilayer architecture. To further assess the potential of the Bi_2_O_2_Se/Bi_2_O_5_Se bilayer for state‐of‐the‐art technology applications, we compared its device performance with that of previously reported systems, as summarized in Table S1. We also considered the energy bandgap and interfacial band alignment of the RRAM devices (Figure S14) to further support the electron transport during the switching process, thus establishing a structural and electrical switching mechanism. It is worth noting that α‐Bi_2_SeO_5_ has recently been identified as a high‐performance van der Waals ferroelectric oxide [34]. While our in situ TEM and EELS analysis confirm that electric‐field‐driven oxidation is the dominant driving force for the observed resistive switching, the intrinsic ferroelectric polarization of α‐Bi_2_SeO_5_ may coexist as a background effect. The potential coupling between ferroelectric domains and the distribution of oxygen vacancies offers a compelling direction for further enhancing synaptic plasticity and switching consistency in Bi_2_O_2_Se/Bi_2_O_5_Se heterostructures.

## 4 Conclusion

We demonstrated that the Bi_2_O_2_Se/Bi_2_O_5_Se bilayer exhibited a controllable and reliable resistive switching behavior using an electric‐field‐driven oxidation process. The complementary roles of the two layers established a stable ionic transport pathway, supporting consistent operation over repeated cycling. Atomic‐scale STEM and in situ TEM analyses directly demonstrated the structural evolution underlying the switching, providing direct evidence of the interfacial dynamic mechanism combined with the demonstrated electrical performance, including a long retention time (12 000 s), endurance (>3 million cycles), and stable multilevel state control. In addition, a variety of pulse measurements that emulated synaptic‐like behavior were achieved. This bilayer architecture provided a promising material for nonvolatile memory and energy‐efficient neuromorphic applications.

## 5 Experimental Section

### 5.1 Sample Preparation

Bi_2_O_2_Se thin films were deposited on 0.5 wt.% Nb‐doped SrTiO_3_ (Nb–STO) substrates using pulsed laser deposition (PLD) with a commercial Bi_2_O_2_Se target. Prior to deposition, the chamber was evacuated to a base pressure of 1 × 10^−6^ Torr. The substrate temperature was increased to 425 °C, and the Bi_2_O_2_Se layer was deposited under an oxygen pressure of 0.1 Torr. After deposition, the oxygen pressure was increased to 100 Torr while maintaining a temperature of 425°C for 1 h to promote crystallinity and thermal oxidation. The sample was then cooled to 25°C at a rate of 0.1 °C/s. This annealing step fully oxidized the Bi_2_O_2_Se layer into Bi_2_O_5_Se. To construct the bilayer structure, the chamber was re‐evacuated to 4 × 10^−6^ Torr, and the substrate was reheated to 425 °C under a 0.1‐Torr oxygen pressure. A second Bi_2_O_2_Se layer was deposited under the same conditions to form a Bi_2_O_2_Se/Bi_2_O_5_Se stacked structure. Finally, a 20‐nm Ti adhesion layer followed by a 70‐nm Au top electrode was deposited by E‐gun evaporation at a rate of 0.5 Å/s. Circular top electrodes (200 µm in diameter) were defined using a metal shadow mask.

### 5.2 Characterizations of the Au/Ti/Bi_2_O_2_Se/Bi_2_O_5_Se/Nb–STO RRAM Devices

TEM lamellar specimens were prepared using a Zeiss Crossbeam 550 focused ion beam (FIB) system, with the thickness controlled to less than 100 nm to ensure high‐resolution imaging (Figure S15). The prepared lamellar samples were subsequently transferred onto Cu grids with glass tips. For in situ TEM observations, the samples were also fabricated using FIB and transferred onto an electrical chip, where Pt contacts were deposited to connect the device electrodes to the chip electrodes. The samples were then loaded into a specialized in situ TEM holder (Protochips FusionAX), allowing electrical biasing during the resistive switching observation. High‐resolution TEM imaging and elemental mapping were performed using a field‐emission TEM instrument (JEOL‐F200, 200 kV) coupled to an Oxford EDS 100 TLE detector. Additionally, atomic‐scale annular dark‐field (ADF)–STEM analysis and oxidation state confirmation were performed using a STEM instrument with a Cs‐corrector (JEOL JEM‐ARM200F) and electron energy loss spectroscopy (GATAN Quantum 965 spectrometer). XPS data were obtained using an X‐ray photoelectron spectrometer (PHI Quantera II).

### 5.3 Electrical Characterization

The electrical properties of the fabricated RRAM devices were characterized using semiconductor parameter analyzers (Keysight B1500A). All electrical measurements were conducted using a biased top electrode and a grounded Nb–STO bottom electrode.

## Conflicts of Interest

The authors declare no conflicts of interest.

## Supporting information




**Supporting File 1**: advs75508‐sup‐0001‐SuppMat.docx.

**Supporting File 2**: advs75508‐sup‐0002‐Movie S1.mp4.

**Supporting File 3**: advs75508‐sup‐0003‐Movie S2.mp4.

**Supporting File 4**: advs75508‐sup‐0004‐Movie S3.mp4.

## Data Availability

The data supporting the findings of this study can be found in the supplementary materials of this article.

## References

[1] J. Park , A. Kumar , Y. Zhou , et al., “Multi‐Level, Forming and Filament Free, Bulk Switching Trilayer Rram for Neuromorphic Computing at the Edge,” Nature Communications 15 (2024): 3492, 10.1038/s41467-024-46682-1. PMC11045755 38664381

[2] M. Prezioso , F. Merrikh‐Bayat , B. D. Hoskins , G. C. Adam , K. K. Likharev , and D. B. Strukov , “Training and Operation of an Integrated Neuromorphic Network Based on Metal‐Oxide Memristors,” Nature 521 (2015): 61–64, 10.1038/nature14441. 25951284

[3] K. U. Mohanan , “Resistive Switching Devices for Neuromorphic Computing: From Foundations to Chip Level Innovations,” Nanomaterials 14 (2024): 527, 10.3390/nano14060527. 38535676 PMC10976006

[4] D. Ielmini and G. Pedretti , “Resistive Switching Random‐Access Memory (Rram): Applications and Requirements for Memory and Computing,” Chemical Reviews 125 (2025): 5584–5625, 10.1021/acs.chemrev.4c00845. 40314431 PMC12203480

[5] D. Ielmini and H.‐S. P. Wong , “In‐Memory Computing with Resistive Switching Devices,” Nature Electronics 1 (2018): 333.

[6] M. Prezioso , M. R. Mahmoodi , F. M. Bayat , et al., “Spike‐Timing‐Dependent Plasticity Learning of Coincidence Detection With Passively Integrated Memristive Circuits,” Nature Communications 9 (2018): 5311, 10.1038/s41467-018-07757-y. PMC6294012 30552327

[7] J. Kang , T. Kim , S. Hu , et al., “Cluster‐Type Analogue Memristor by Engineering Redox Dynamics for High‐Performance Neuromorphic Computing,” Nature Communications 13 (2022): 4040, 10.1038/s41467-022-31804-4. PMC9279478 35831304

[8] L. Wang , W. Liao , S. L. Wong , et al., “Artificial Synapses Based on Multiterminal Memtransistors for Neuromorphic Application,” Advanced Functional Materials 29 (2019): 1901106, 10.1002/adfm.201901106.

[9] L. Chua , “Resistance Switching Memories Are Memristors,” Applied Physics A 102 (2011): 765–783, 10.1007/s00339-011-6264-9.

[10] J. Y. Chen , C. W. Huang , C. H. Chiu , Y. T. Huang , and W. W. Wu , “Switching Kinetic of VCM‐Based Memristor: Evolution and Positioning of Nanofilament,” Advanced Materials 27 (2015): 5028–5033, 10.1002/adma.201502758. 26193454

[11] C.‐H. Wang , H.‐Y. Lo , C.‐W. Huang , J.‐Y. Chen , and W.‐W. Wu , “Enhanced Resistive Switching Performance and Structural Evolution of NiO/Nb_2_O_5_− X Bilayer Memristive Device,” Journal of Alloys and Compounds 983 (2024): 173889, 10.1016/j.jallcom.2024.173889.

[12] M.‐H. Yang , C.‐H. Wang , Y.‐H. Lai , et al., “Antiferroelectric Heterostructures Memristors With Unique Resistive Switching Mechanisms and Properties,” Nano Letters 24 (2024): 11482–11489, 10.1021/acs.nanolett.4c02705. 39158148

[13] Y. Yang , P. Gao , S. Gaba , T. Chang , X. Pan , and W. Lu , “Observation of Conducting Filament Growth in Nanoscale Resistive Memories,” Nature Communications 3 (2012): 732, 10.1038/ncomms1737. 22415823

[14] V. R. Nallagatla , T. Heisig , C. Baeumer , et al., “Topotactic Phase Transition Driving Memristive Behavior,” Advanced Materials 31 (2019): 1903391, 10.1002/adma.201903391. 31441160

[15] Y.‐J. Chen , H.‐Y. Lo , C.‐C. Chiu , et al., “Atomic‐Scale Phase Transformation in Perovskite LaCoO x Resistive Switching Memristive Devices,” Small Structures 5 (2024): 2400019, 10.1002/sstr.202400019.

[16] W. Li , X. Gong , Z. Yu , et al., “Approaching the Quantum Limit in Two‐Dimensional Semiconductor Contacts,” Nature 613 (2023): 274–279, 10.1038/s41586-022-05431-4. 36631650

[17] A. K. Geim and I. V. Grigorieva , “Van der Waals heterostructures,” Nature 499 (2013): 419–425, 10.1038/nature12385. 23887427

[18] K. Kang , S. Xie , L. Huang , et al., “High‐Mobility Three‐Atom‐Thick Semiconducting Films With Wafer‐Scale Homogeneity,” Nature 520 (2015): 656–660, 10.1038/nature14417. 25925478

[19] S. J. Kim , H.‐J. Lee , C.‐H. Lee , and H. W. Jang , “2d Materials‐Based 3d Integration for Neuromorphic Hardware,” npj 2D Materials and Applications 8 (2024): 70.

[20] S. S. Teja Nibhanupudi , A. Roy , D. Veksler , et al., “Ultra‐Fast Switching Memristors Based on Two‐Dimensional Materials,” Nature Communications 15 (2024): 2334, 10.1038/s41467-024-46372-y. PMC10940724 38485722

[21] H. Zhou , S. Li , K.‐W. Ang , and Y.‐W. Zhang , “Recent Advances in in‐Memory Computing: Exploring Memristor and Memtransistor Arrays With 2d Materials,” Nano‐Micro Letters 16 (2024): 121, 10.1007/s40820-024-01335-2. 38372805 PMC10876512

[22] X. Zhu , D. Li , X. Liang , and W. D. Lu , “Ionic Modulation and Ionic Coupling Effects in Mos2 Devices for Neuromorphic Computing,” Nature Materials 18 (2019): 141–148, 10.1038/s41563-018-0248-5. 30559410

[23] J. Wu , H. Yuan , M. Meng , et al., “High Electron Mobility and Quantum Oscillations in Non‐Encapsulated Ultrathin Semiconducting Bi_2_O_2_Se,” Nature Nanotechnology 12 (2017): 530–534, 10.1038/nnano.2017.43. 28369044

[24] H. Tao , T. Wang , D. Li , J. Xing , and G. Li , “Preparation, Properties, and Applications of Bi_2_O_2_Se Thin Films: A Review,” Journal of Semiconductors 44 (2023): 031001, 10.1088/1674-4926/44/3/031001.

[25] C. Tan , M. Yu , J. Tang , et al., “2d Fin Field‐Effect Transistors Integrated With Epitaxial High‐K Gate Oxide,” Nature 616 (2023): 66–72, 10.1038/s41586-023-05797-z. 36949195

[26] S. Wang , K. Liang , H. Zhao , et al., “Electronic Structure Formed by Y_2_O_3_‐Doping in Lithium Position Assists Improvement of Charging‐Voltage for High‐Nickel Cathodes,” Nature Communications 16 (2025), 10.1038/s41467-024-52768-7. PMC11697207 39746907

[27] J. Yin , Z. Tan , H. Hong , et al., “Ultrafast and Highly Sensitive Infrared Photodetectors Based on Two‐Dimensional Oxyselenide Crystals,” Nature Communications 9 (2018): 3311, 10.1038/s41467-018-05874-2. PMC6098096 30120240

[28] Z. Wang , L. Liu , K. Zhai , et al., “An Ultrasensitive Plasmonic Sensor Based on 2D Ferroelectric Bi_2_O_2_ Se,” Small 19 (2023): 2303026, 10.1002/smll.202303026. 37394706

[29] T. Li , T. Tu , Y. Sun , et al., “A Native Oxide High‐κ Gate Dielectric for Two‐Dimensional Electronics,” Nature Electronics 3 (2020): 473–478, 10.1038/s41928-020-0444-6.

[30] Y. Zhang , J. Yu , R. Zhu , et al., “A Single‐Crystalline Native Dielectric for Two‐Dimensional Semiconductors With an Equivalent Oxide Thickness Below 0.5 nm,” Nature Electronics 5 (2022): 643–649, 10.1038/s41928-022-00824-9.

[31] Y. Zhao , Z. Lou , J. Hu , et al., “Scalable Layer‐Controlled Oxidation of Bi_2_O_2_ Se for Self‐Rectifying Memristor Arrays With sub‐pA Sneak Currents,” Advanced Materials 36 (2024): 2406608, 10.1002/adma.202406608. 39246123

[32] Y. Xia , J. Wang , R. Chen , et al., “2d Heterostructure of Bi_2_O_2_Se /Bi_2_SeOx Nanosheet for Resistive Random Access Memory,” Advanced Electronic Materials 8 (2022): 2200126, 10.1002/aelm.202200126.

[33] T. Tu , Y. Zhang , T. Li , et al., “Uniform High‐K Amorphous Native Oxide Synthesized by Oxygen Plasma for Top‐Gated Transistors,” Nano Letters 20 (2020): 7469–7475, 10.1021/acs.nanolett.0c02951. 32881534

[34] Q. Wu , Z. Li , B. Han , et al., “Wafer‐Scale Ultrathin and Uniform Van Der Waals Ferroelectric Oxide,” Science 391 (2026): adz1655, 10.1126/science.adz1655. 41610232

[35] Z. Dong , Q. Hua , J. Xi , et al., “Ultrafast and Low‐Power 2d Bi_2_O_2_Se Memristors for Neuromorphic Computing Applications,” Nano Letters 23 (2023): 3842–3850, 10.1021/acs.nanolett.3c00322. 37093653

[36] J. Wu , C. Tan , Z. Tan , et al., “Controlled Synthesis of High‐Mobility Atomically Thin Bismuth Oxyselenide Crystals,” Nano Letters 17 (2017): 3021–3026, 10.1021/acs.nanolett.7b00335. 28398056

[37] Y. Y. Hsieh , Y. C. Chuang , and H. Y. Tuan , “Unraveling Dual Mechanisms in Quasi‐Layered Bi_2_O_2_ Se via Defect Modulation for High‐Performance Aqueous Zn‐Ion Batteries,” Advanced Functional Materials 34 (2024): 2406975, 10.1002/adfm.202406975.

[38] T. Kim , T. Vogel , E. Piros , et al., “Oxide Thickness‐Dependent Resistive Switching Characteristics of Cu/HfO_2_/Pt Ecm Devices,” Applied Physics Letters 122 (2023): 023502, 10.1063/5.0124781.

[39] P. Zhang , Y. S. Ang , A. L. Garner , Á. Valfells , J. W. Luginsland , and L. K. Ang , “Space–charge limited current in nanodiodes: Ballistic, collisional, and dynamical effects,” Journal of Applied Physics 129 (2021): 100902, 10.1063/5.0042355.

[40] K.‐H. Chen , R. Zhang , T.‐C. Chang , et al., “Hopping Conduction Distance Dependent Activation Energy Characteristics of Zn: SiO_2_ Resistance Random Access Memory Devices,” Applied Physics Letters 102 (2013): 133503, 10.1063/1.4799655.

[41] D. Ielmini , F. Nardi , and C. Cagli , “Physical Models of Size‐Dependent Nanofilament Formation and Rupture in NiO Resistive Switching Memories,” Nanotechnology 22 (2011): 254022, 10.1088/0957-4484/22/25/254022. 21572207

[42] Y. N. Zhong , T. Wang , X. Gao , J. L. Xu , and S. D. Wang , “Synapse‐Like Organic Thin Film Memristors,” Advanced Functional Materials 28 (2018): 1800854, 10.1002/adfm.201800854.

[43] D. Kim , J. Kim , and S. Kim , “Enhancement of Resistive and Synaptic Characteristics in Tantalum Oxide‐Based Rram by Nitrogen Doping,” Nanomaterials 12 (2022): 3334, 10.3390/nano12193334. 36234461 PMC9565720

[44] M. F. Bear and R. C. Malenka , “Synaptic Plasticity: LTP and LTD,” Current Opinion in Neurobiology 4 (1994): 389–399, 10.1016/0959-4388(94)90101-5. 7919934

[45] M. Ismail , H. Abbas , A. Sokolov , C. Mahata , C. Choi , and S. Kim , “Emulating Synaptic Plasticity and Resistive Switching Characteristics Through Amorphous Ta_2_O_5_ Embedded Layer for Neuromorphic Computing,” Ceramics International 47 (2021): 30764–30776, 10.1016/j.ceramint.2021.07.257.

[46] S. Li , M.‐E. Pam , Y. Li , et al., “Wafer‐Scale 2D Hafnium Diselenide Based Memristor Crossbar Array for Energy‐Efficient Neural Network Hardware,” Advanced Materials 34 (2022): 2103376, 10.1002/adma.202103376. 34510567

[47] J. Park , Y. Jang , J. Lee , S. An , J. Mok , and S.‐Y. Lee , “Synaptic Transistor Based on In‐Ga‐Zn‐O Channel and Trap Layers With Highly Linear Conductance Modulation for Neuromorphic Computing,” Advanced Electronic Materials 9 (2023): 2201306, 10.1002/aelm.202201306.

[48] T. J. Frankcombe and Y. Liu , “Interpretation of Oxygen 1s X‐Ray Photoelectron Spectroscopy of ZnO,” Chemistry of Materials 35 (2023): 5468–5474, 10.1021/acs.chemmater.3c00801.

[49] J. H. Jang , H.‐S. Jung , J. H. Kim , S. Y. Lee , C. S. Hwang , and M. Kim , “Investigation of Oxygen‐Related Defects and the Electrical Properties of Atomic Layer Deposited HfO_2_ Films Using Electron Energy‐Loss Spectroscopy,” Journal of Applied Physics 109 (2011): 023718, 10.1063/1.3544039.

[50] H. Kurata and C. Colliex , “Electron‐Energy‐Loss Core‐Edge Structures in Manganese Oxides,” Physical Review B 48 (1993): 2102–2108, 10.1103/PhysRevB.48.2102. 10008600

[51] X. Ding , M. Li , P. Chen , et al., “Bi_2_O_2_Se: A Rising Star for Semiconductor Devices,” Matter 5 (2022): 4274.

[52] W. Chen , R. Zhang , R. Zheng , and B. Liu , “Out‐of‐Plane Resistance Switching of 2D Bi_2_O_2_ Se at the Nanoscale,” Advanced Functional Materials 31 (2021): 2105795, 10.1002/adfm.202105795.

[53] X. Huang , C.‐Y. Niu , J. Zhang , A. Wang , Y. Jia , and Y. Song , “Strain‐Tunable Electronic Structure, Optical Response, and High Electron Mobility of Bi_2_O_2_Se Crystals,” APL Materials 7 (2019): 081110, 10.1063/1.5108853.

