# Supplementary material for: Epitaxial Bi2O2Se/Bi2O5Se Thin Films: Revealing Electric‐Field‐Driven Oxidation and Resistive Switching Dynamics for Advanced Memory Devices
Source: Adv Sci (Weinh). 2026 May 7;13(41):e75508. doi: 10.1002/advs.75508 (PMC13335449; doi:10.1002/advs.75508)
Supplement: Supplementary file 1 — Supporting File 1: advs75508‐sup‐0001‐SuppMat.docx. [file ADVS-13-e75508-s003.docx]

Supporting Information

**Epitaxial Bi_2_O_2_Se/Bi_2_O_5_Se Thin Films: Revealing Electric-Field-Driven Oxidation and Resistive Switching Dynamics for Advanced Memory Devices.**

*Yen-Jung Chen, Yong-Jyun Wang, Zi-Qin Hong, Chien-Hua Wang, Yi-Ning Wang, Jia De Cheng, Chun-Wei Huang, Ying-Hao Chu and Wen-Wei Wu**

List of contents

Supplementary Note 1. Fabrication of Au/Ti/Bi_2_O_2_Se/α-Bi_2_O_5_Se/Nb–STO RRAM devices.

Supplementary Note 2. Optical and Raman identification of bilayer and single-layer Bi_2_O_5_Se devices.

Supplementary Note 3. Electrical characteristics and switching mechanisms of the RRAM devices.

Supplementary Note 4. Microstructural evolution of the RRAM devices demonstrated by STEM and TEM.

Supplementary Note 5. Chemical and interfacial evolution determined by XPS and EELS analyses.

Supplementary Note 6. Mechanism model for resistive switching.

Supplementary Note 7. Experimental setup for *in situ* TEM measurements.

Figure S1. Fabrication of the Au/Ti/Bi_2_O_2_Se/Bi_2_O_5_Se/Nb–STO RRAM devices.

Figure S2. Optical microscopy images and Raman spectra of the Bi_2_O_2_Se/Bi_2_O_5_Se bilayer and Bi_2_O_5_Se single layer.

Figure S3. Pure Bi_2_O_5_Se dielectric layer RRAM devices.

Figure S4. *I–V* characteristics and multistate switching behavior of the RRAM devices.

Figure S5. The *I–V* fitting curves analysis for resistive switching mechanisms.

Figure S6. Temperature impact on retention and switching characteristics of Au/Ti/Bi_2_O_2_Se/Bi_2_O_5_Se/Nb–STO devices.

Figure S7. Synaptic behaviors and current responses of the Au/Ti/Bi_2_O_2_Se/Bi_2_O_5_Se/Nb-STO devices.

Figure S8. Cross-sectional STEM–EDS elemental mapping of the RRAM devices before and after electrical measurements.

Figure S9. Wide-field TEM image and STEM image of the cycled device.

Figure S10. Microstructural evolution of the Bi_2_O_2_Se/Bi_2_O_5_Se bilayer and EDS point analysis before and after electrical measurements.

Figure S11. High-resolution XPS spectra of the pristine and cycled RRAM devices.

Figure S12. XPS spectra of Bi 4f for the pristine and cycled devices.

Figure S13. STEM–EELS analysis of the interfacial evolution in the RRAM device.

Figure S14. Energy-band diagrams and resistive switching mechanism.

Figure S15. Experimental setup for in situ TEM analysis using FIB system and E-chip holder.

Table S1. Comparison of the Bi_2_O_2_Se/Bi_2_O_5_Se heterostructure RRAM device with previous memristors.

**Supplementary Note 1. Fabrication of Au/Ti/Bi_2_O_2_Se/α-Bi_2_O_5_Se/Nb–STO RRAM devices.**

This note provides a step-by-step description of the fabrication of the bilayer RRAM devices (Figure S1a), complementing the experimental details presented in the Methods section.

a. The initial Bi_2_O_2_Se layer was deposited on an Nb-doped SrTiO_3_ substrate for subsequent oxide formation.

b. The Bi_2_O_2_Se layer was then fully oxidized into the thermodynamically stable bismuth selenite α-Bi_2_O_5_Se phase through an oxygen annealing process, which acted as the bottom oxide layer.

c. To construct the bilayer architecture, a second Bi_2_O_2_Se layer was deposited on top of the bottom oxide layer under identical growth conditions. This step was omitted for RRAM devices consisting of a single α-Bi_2_O_5_Se layer.

d. Ti/Au top electrodes were deposited using a circular shadow mask to define individual devices.

By following these deposition steps, we achieved a smooth, epitaxial Bi_2_O_2_Se/α-Bi_2_O_5_Se bilayer stack on the Nb**–**STO substrate. A representative STEM image, provided in Figure S1b, reveals a well-defined interface and continuous film coverage across a large area, demonstrating the superior crystallinity of the bilayer.

**Supplementary Note 2. Optical and Raman identification of bilayer and single-layer Bi_2_O_5_Se devices.**

Figure S2a,b show a clear optical contrast between the bilayer and single-layer devices originating from the distinct optical properties of the two oxide phases. Bi_2_O_5_Se exhibited a widened bandgap and was optically transparent in the visible range, absorbing light primarily below approximately 320 nm^[1]^, which allowed the underlying Nb–STO substrate to dominate the observed contrast. By contrast, the presence of a top Bi_2_O_2_Se layer in the bilayer device resulted in a lighter appearance. The optical images were acquired under identical imaging conditions, including the same red-green-blue (RGB) balance, contrast, and brightness settings, ensuring that the observed color contrast reflected the intrinsic differences between the two oxide phases rather than imaging artifacts. The Raman spectroscopy further supported this distinction (Figure S2c,d). The bilayer device exhibited a characteristic vibrational mode at approximately 159 cm⁻¹ associated with Bi_2_O_2_Se^[2-3]^, whereas the single-layer Bi_2_O_5_Se device showed a Raman peak at approximately 250 cm⁻¹ ^[4]^. These spectral features were consistent with the previously reported Raman signatures of Bi_2_O_2_Se and Bi_2_O_5_Se, supporting phase identification.

For comparison, RRAM devices based on a single Bi_2_O_5_Se dielectric layer were fabricated and characterized (Figure S3). XRD analysis confirmed the formation of crystalline Bi_2_O_5_Se with a diffraction peak at 32.6° indexed to the (004) plane (Figure S3a). Direct current (DC) *I–V* measurements showed stable switching over 400 cycles. However, compared with the bilayer devices, the single-layer Bi_2_O_5_Se RRAM devices required higher operating voltages and exhibited lower on/off ratios (Figure S3b). STEM imaging verified that the growth route described in Supplementary Note 1 successfully converted the entire Bi_2_O_2_Se layer into Bi_2_O_5_Se after annealing and enabled epitaxial growth on the Nb–STO substrate (Figure S3c). The XPS analysis demonstrated a dominant Se 3d peak at 59.3 eV, corresponding to Se^4+^, with no detectable Se^2-^ signal, indicating the complete oxidation of Se in the single Bi_2_O_5_Se dielectric layer (Figure S3d).

**Supplementary Note 3. Electrical characteristics and switching mechanisms of the RRAM devices.**

The electrical switching behaviors of the Au/Ti/Bi_2_O_2_Se/Bi_2_O_5_Se/Nb–STO devices were investigated by DC and pulse measurements (Figure S4–7). The *I–V* characteristics measured over 420 consecutive DC switching cycles demonstrated a large on/off ratio (Figure S4a). Notably, a gradual increase in the HRS was observed with repeated voltage sweeps. Multilevel resistive switching was achieved by controlling the SET compliance current and RESET voltage (Figure S4b–d). Increasing the compliance current from 1 to 15 mA systematically tuned the LRS from approximately 4 × 10^5^ to approximately 5 × 10^3^ Ω, yielding clearly distinguishable resistance levels with stable retention over 1000 s. Similarly, increasing the RESET voltage from 4 to 5.5 V produced progressively higher HRS levels, ranging from approximately 1 × 10^5^ to approximately 3 × 10^6^ Ω, which remained stable for at least 600 s. These results demonstrated the reliable and controllable multilevel programming of both the LRS and HRS, with good retention characteristics.

To clarify the resistive switching behavior, several conduction models were considered, including Ohmic conduction, space-charge-limited current (SCLC), Schottky emission, and Poole–Frenkel (PF) emission. In Figure S5, ohmic conduction is characterized by a linear current–voltage relationship (I ∝ V), corresponding to a slope of approximately 1 in ln(I)–ln(V) plots^[5]^. The SCLC dominates when the density of the injected carriers exceeds that of the thermally generated carriers in the bulk. In the trap-free regime, the current follows I ∝ V^2^. As the applied voltage increases, a trap-controlled SCLC region emerges, characterized by a steeper slope, until the trap-filled limit voltage (V_TFL_) is reached. Beyond V_TFL_, the traps are filled, and the transport reverts to trap-free SCLC behavior^[6-7]^. Schottky emission occurs when carriers thermionically emit over an interfacial energy barrier under the influence of an applied electric field. This mechanism can be identified by a linear relationship between ln(I) and V^1/2^, indicating that charge transport becomes interface-limited after partial rupture of the conductive filament^[8]^. Poole–Frenkel (PF) emission involves the field-enhanced thermal excitation of trapped carriers into the conduction band. In PF conduction, the slope of the ln(I/V)–V^1/2^ plot is expected to be approximately twice that of Schottky emission^[9]^. In this study, the extracted slopes did not satisfy this criterion, thereby excluding PF emission as the dominant conduction mechanism.

To evaluate the thermal stability, the devices were held at fixed temperatures, and the HRS and LRS were measured for 100 s, as shown in Figure S6a. Both resistance states remained stable over the measured duration, indicating reliable operation across the tested temperature range. The temperature-dependent resistance of the HRS was further analyzed using Arrhenius fitting (Figure S6b). Here, R_0_ was defined as the resistance measured at 298 K, and a linear dependence of ln (R_T_/R_0_) on 1/T was observed. This behavior supports defect-mediated conduction, in agreement with the trap-controlled SCLC behavior identified from the ln(I)–ln(V) plot. By contrast, the LRS exhibited a weak dependence of R_T_/R_0_ on T (Figure S6c), and it did not follow Arrhenius behavior. This weak temperature dependence is consistent with the Ohmic-like conduction observed in the ln(I)–ln(V) plots.

Pulse measurements were performed to evaluate the synaptic plasticity of the RRAM devices (Figure S7). The current response increased with increasing pulse amplitude, indicating the progressive strengthening of the conductive pathway under stronger electrical stimulation (Figure S7a). The long-term potentiation (LTP) and long-term depression (LTD) behaviors were investigated by applying 40 consecutive potentiation and depression pulses (Figure S7b). As illustrated in Figure S7c, the SET and RESET pulses with amplitudes of -1.8 V and 2.5 V, respectively, both use a 100 ns pulse width and a 0.1 V read voltage. Near-linear LTP/LTD characteristics (Figure S7d) could be achieved by increasing the pulse amplitude in small steps ( -1.5 to -3.5 V for potentiation and 3.0 to 5.0 V for depression, with a step size of 0.1 V), which enabled more uniform conductance updates that were essential for accurate synaptic weight modulation in neuromorphic computing applications. Increasing either the potentiation voltage (from −1.8 to −3.5 V) or pulse width (from 100 ns to 10 μs) resulted in enhanced conductance modulation (Figure S7e, f). These results indicated that the synaptic weight could be effectively tuned by adjusting either the pulse amplitude or the pulse width.

**Supplementary Note 4. Microstructural evolution of the RRAM devices demonstrated by STEM and TEM.**

Atomic-resolution STEM imaging, cross-sectional STEM–EDS elemental mapping, EDS point analysis, and TEM observations were performed to investigate the microstructural evolution of the RRAM devices before and after the electrical measurements (Figure S8–10). STEM–EDS elemental mapping demonstrated uniform elemental distributions in both the pristine and cycled devices, with no detectable interdiffusion or compositional loss (Figure S8). This observation suggested that the resistive switching process was not driven by electrode diffusion but was instead associated with intrinsic defect-related conduction. Wide-field TEM and STEM observations of the cycled device (Figure S9) confirmed that the bilayer structure was preserved after repeated electrical operations. The boundary between the stoichiometric BO5S and oxygen-deficient BO5-xS regions is highlighted by the yellow dashed lines. Local interfacial roughening was observed in certain regions, which was attributed to oxygen migration toward the upper BO2S layer. In addition, the vertical structural distortion across the BO2S layer (Figure S9b) implied the formation of a defect-mediated conduction pathway. Detailed atomic-scale observations and EDS point analyses (Figure S10) demonstrated lattice expansion and rearrangement of the local coordination framework at the interface of the BO2S/BO5S layer. EDS point analyses acquired at positions 1–4, as indicated in the initial and cycled states, showed a reduced oxygen atomic percentage after electrical treatment, indicating the formation of oxygen-deficient channels.

**Supplementary Note 5. Chemical and interfacial evolution determined by XPS and EELS analyses.**

Depth-resolved XPS analysis was employed to examine the evolution of the chemical state across the Bi_2_O_2_Se/Bi_2_O_5_Se stack before and after electrical cycling (Figure S11). The XPS depth profiles (Figure S11a,b) show a clear compositional gradient across the heterostructure. The waterfall spectra (Figure S11c,d) were acquired sequentially from the top electrode toward the substrate, corresponding to positions 1–5 in the pristine state and positions 6–10 after electrical cycling. After cycling, the Bi^3+^ component became more pronounced with the O 1s peak located at a higher binding energy, indicating a stronger oxygen coordination environment. Additionally, the Se 3d spectra exhibit a significant feature at approximately 58 eV, corresponding to the oxidized Se^4+^ states, suggesting an increased thickness of the oxidized Bi_2_O_5_Se layer (positions 8 and 9). The signals near approximately 60 eV originate from the Ti 3s layer^[10]^. By comparison, the Ti 2p signals are confined to Ti^0^ near the top electrode and Ti^4+^ near the Nb-STO substrate, indicating that no Ti diffused into the switching layer after electrical cycling.

In addition to the XPS analysis of Se 3d and O 1s discussed in the main text, the chemical states of Bi were examined. The Bi 4f spectra exhibit a clear spin–orbit doublet corresponding to Bi^3+^ in both the pristine and cycled states. In Figure S12, the upper Bi_2_O_2_Se region remains unchanged after cycling, with the Bi^3+^ component at approximately 158.4 eV (Bi 4f_7/2_). By contrast, the Bi 4f doublet in the bottom layer has shifted slightly toward a higher binding energy (approximately 158.6 to 158.8 eV), indicating enhanced oxygen coordination and local structural relaxation within the Bi–O–Se framework. No Bi^5+^ feature is observed, suggesting that the oxidation of Bi_2_O_2_Se to Bi_2_O_5_Se occurred without any Bi valence change.

STEM–EELS line-scan analyses further demonstrated the interfacial evolution of the devices in different resistance states (Figure S13). As shown in Figure S13d, the atomic percentages of O, Se, and Ti were analyzed because the Bi M-edge lies at a higher energy and is not reliably detectable under the current EELS conditions. In the pristine state, an increased O signal is observed in the bottom layer without a detectable Ti contribution, confirming that the signal originated from the oxidized BO5S film rather than Nb–STO substrate. For the cycled-HRS and cycle-LRS systems, the analysis focused on the O intensity distribution. The bottom BO5S region remained O-rich in the HRS, whereas a significant dip in the O signal is observed in the bottom **BO5-xS** region in the LRS. The black dashed lines indicate the region boundaries defined by comparing the EELS line-scan thicknesses with the cross-sectional STEM images.

**Supplementary Note 6. Mechanism model for resistive switching.**

Figure S14 shows the proposed energy-band diagrams and resistive switching mechanism of the Bi_2_O_2_Se/Bi_2_O_5_Se bilayer RRAM device. The band alignment was constructed based on previously reported bandgaps, where Bi_2_O_2_Se exhibited a narrow indirect bandgap (approximately 0.8 eV), while Bi_2_O_5_Se exhibited a much wider bandgap (approximately 3.9 eV)^[1, 11-12]^. After contact, band bending occurred at the interfaces to align the Fermi levels, thus establishing asymmetric carrier-injection conditions across the heterostructure. During the SET process (Figure S14c), electrons were injected from Ti into Bi_2_O_2_Se, which gradually filled the traps in Bi_2_O_5_Se, leading to space-charge-limited conduction (SCLC). As the traps were gradually filled, conductive paths were established, and the device switched from the HRS to LRS, exhibiting a nearly Ohmic conduction. During the RESET process (Figure S14d), electrons were driven from the Nb–STO bottom electrode toward the Ti electrode. At a low bias, the transport remained Ohmic-like, suggesting that the conductive paths were still partially intact. As the applied bias increased, the conduction mechanism transitioned to Schottky-like emission across the Bi_2_O_5_Se/Nb–STO interface, corresponding to the partial rupture of the conductive paths. Upon sweeping the bias back to 0 V, the conduction gradually relaxed to an Ohmic-like behavior. These transport processes were inferred from the ln(I)–ln(V) analysis presented in Figure S5.

**Supplementary Note 7. Experimental setup for *in situ* TEM measurements.**

Figure S15a–e illustrate the preparation of cross-sectional lamellae using a focused ion beam (FIB) system. A 10-μm length Pt protective layer was first deposited on the device surface to prevent damage during cross-sectional milling. Trench milling and subsequent thinning were performed to reduce the lamellar thickness to less than 100 nm, enabling high-resolution TEM observations. The prepared lamellae were then transferred onto an E-chip using a glass tip. Two Pt leads were individually deposited to connect the top electrode and Nb–STO substrate to the E-chip electrodes. During biasing, the applied voltage was delivered vertically across the switching layer from the top electrode to the grounded Nb–STO substrate. This current path mimicked the out-of-plane electrical configuration used in the bulk RRAM measurements.

**
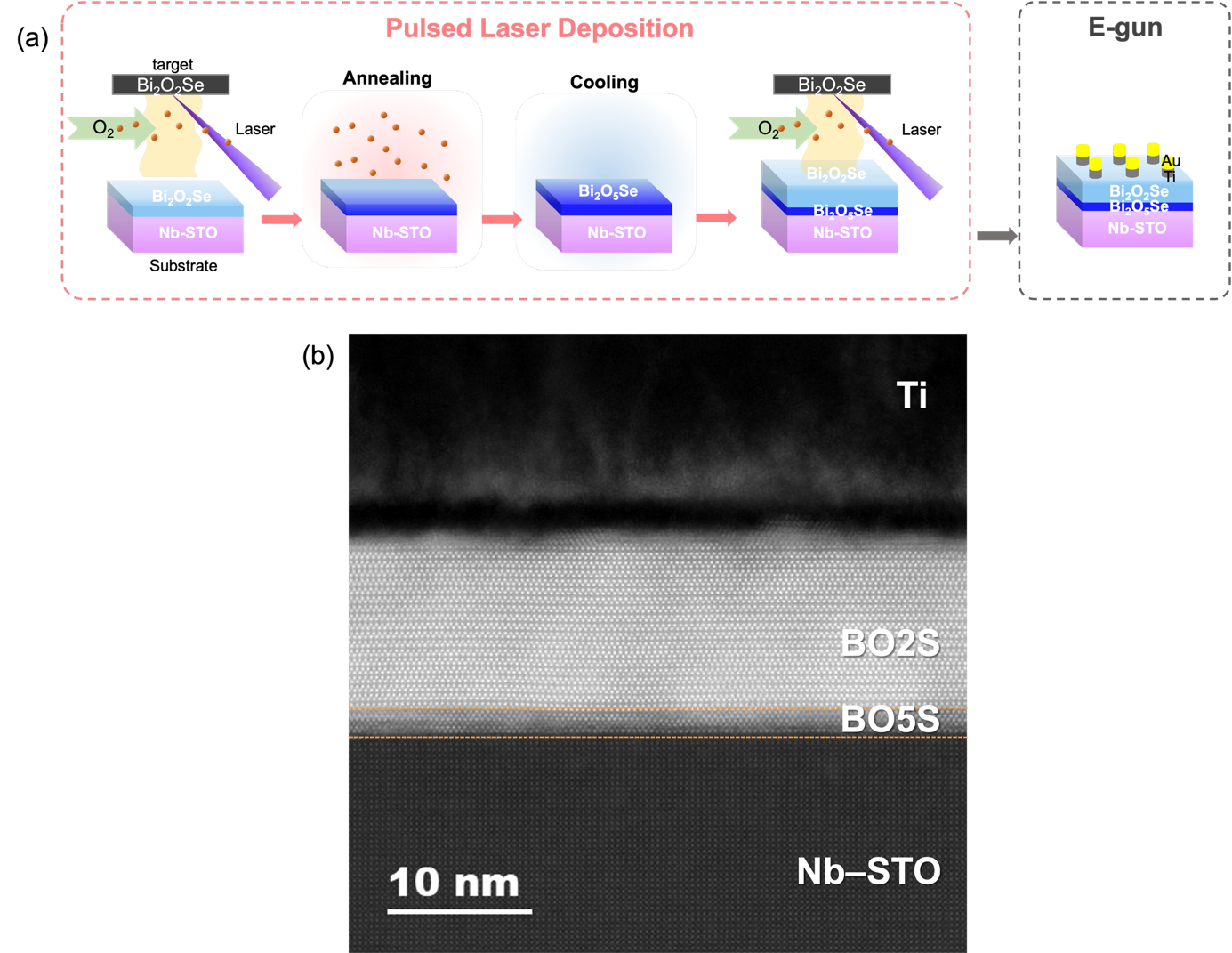
**

**Figure S1.** **Fabrication of Au/Ti/Bi_2_O_2_Se/α-Bi_2_O_5_Se/Nb–STO RRAM devices.** (a)Process for fabricating RRAM devices. (b) Cross-sectional STEM image of the device stack after deposition, showing the well-defined interfaces between layers.

**
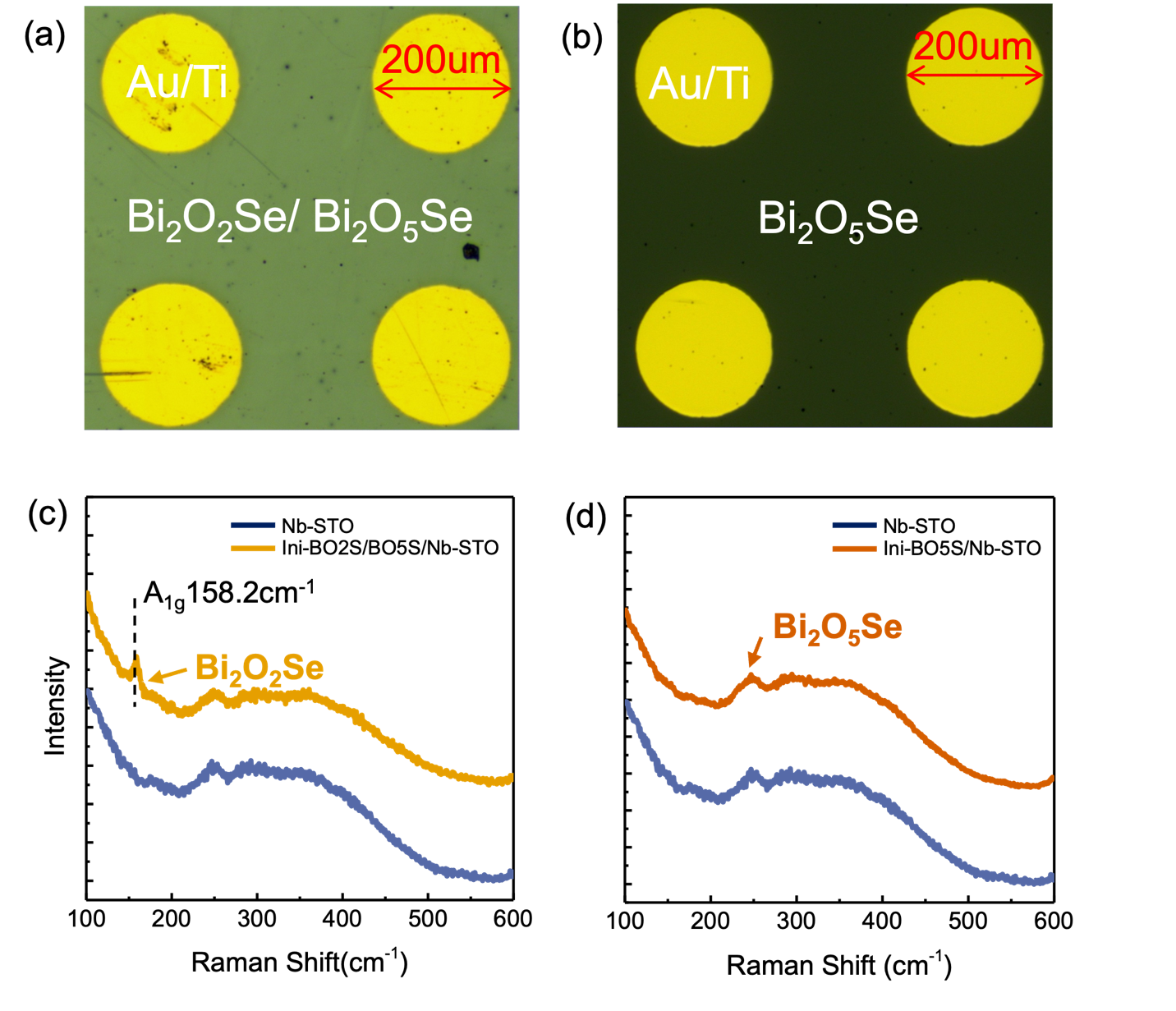
**

**Figure S2. Optical microscopy images and Raman spectra of the Bi_2_O_2_Se/Bi_2_O_5_Se bilayer and Bi_2_O_5_Se single layer.**

(a) Optical microscopy image of the Au/Ti/Bi_2_O_2_Se/Bi_2_O_5_Se/Nb–STO device. (b) Optical microscopy image of the Au/Ti/Bi_2_O_5_Se/Nb–STO device. (c) Raman spectra collected from the Bi_2_O_2_Se/Bi_2_O_5_Se/Nb–STO region and bare Nb–STO substrate. (d) Raman spectra collected from the Bi_2_O_5_Se/Nb–STO region and bare Nb–STO substrate.


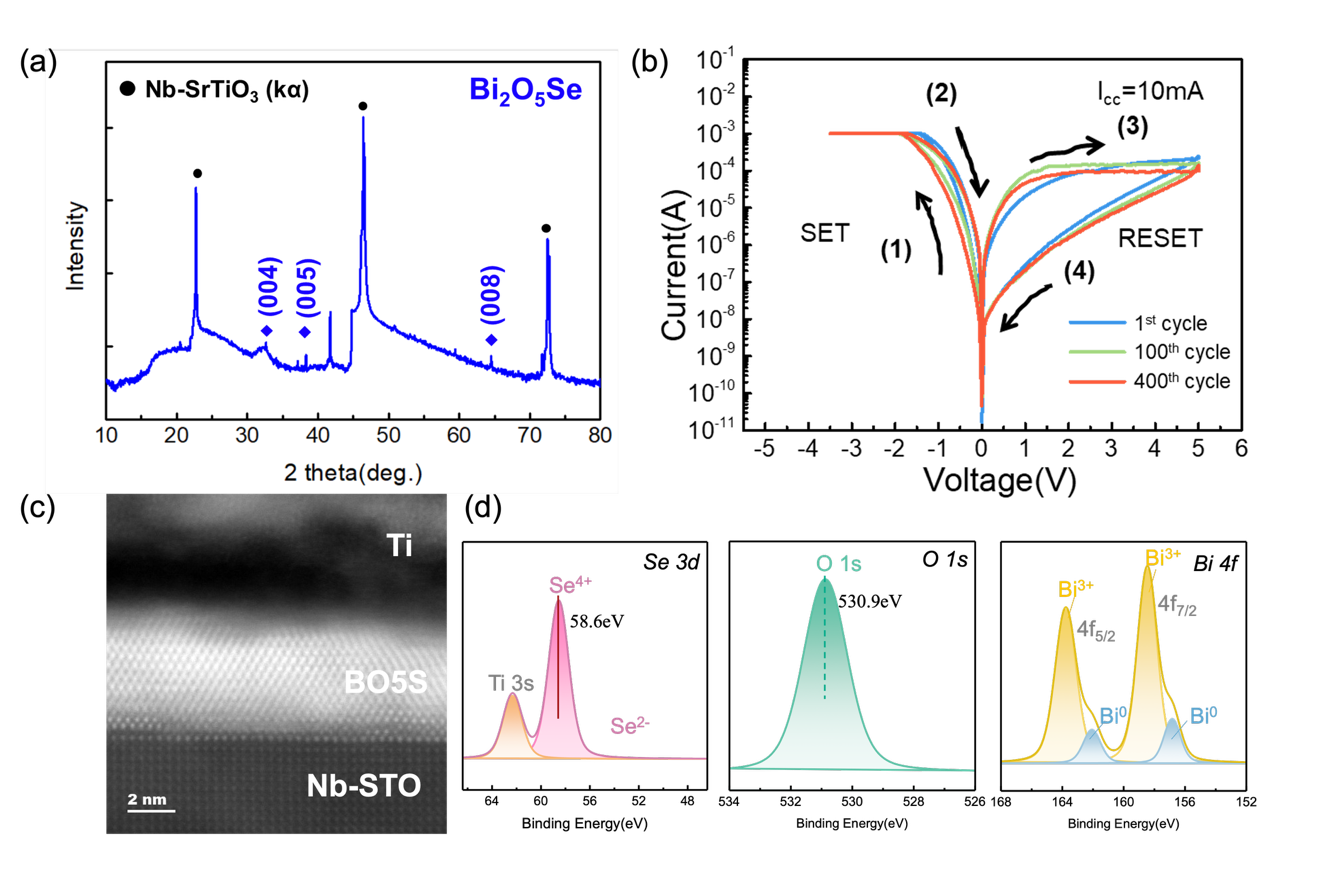


**Figure S3. Pure Bi_2_O_5_Se dielectric layer RRAM devices.**

(a) XRD pattern of the Bi_2_O_5_Se/Nb–STO devices. (b) *I–V* characteristics of 400 consecutive DC switching cycles measured on the Au/Ti/Bi_2_O_5_Se/Nb–STO device, with a SET voltage of -2 V and RESET voltage of 5 V. (c) Cross-sectional STEM image of the single Bi_2_O_5_Se dielectric layer. (d) High-resolution XPS spectrum of the Bi_2_O_5_Se film.


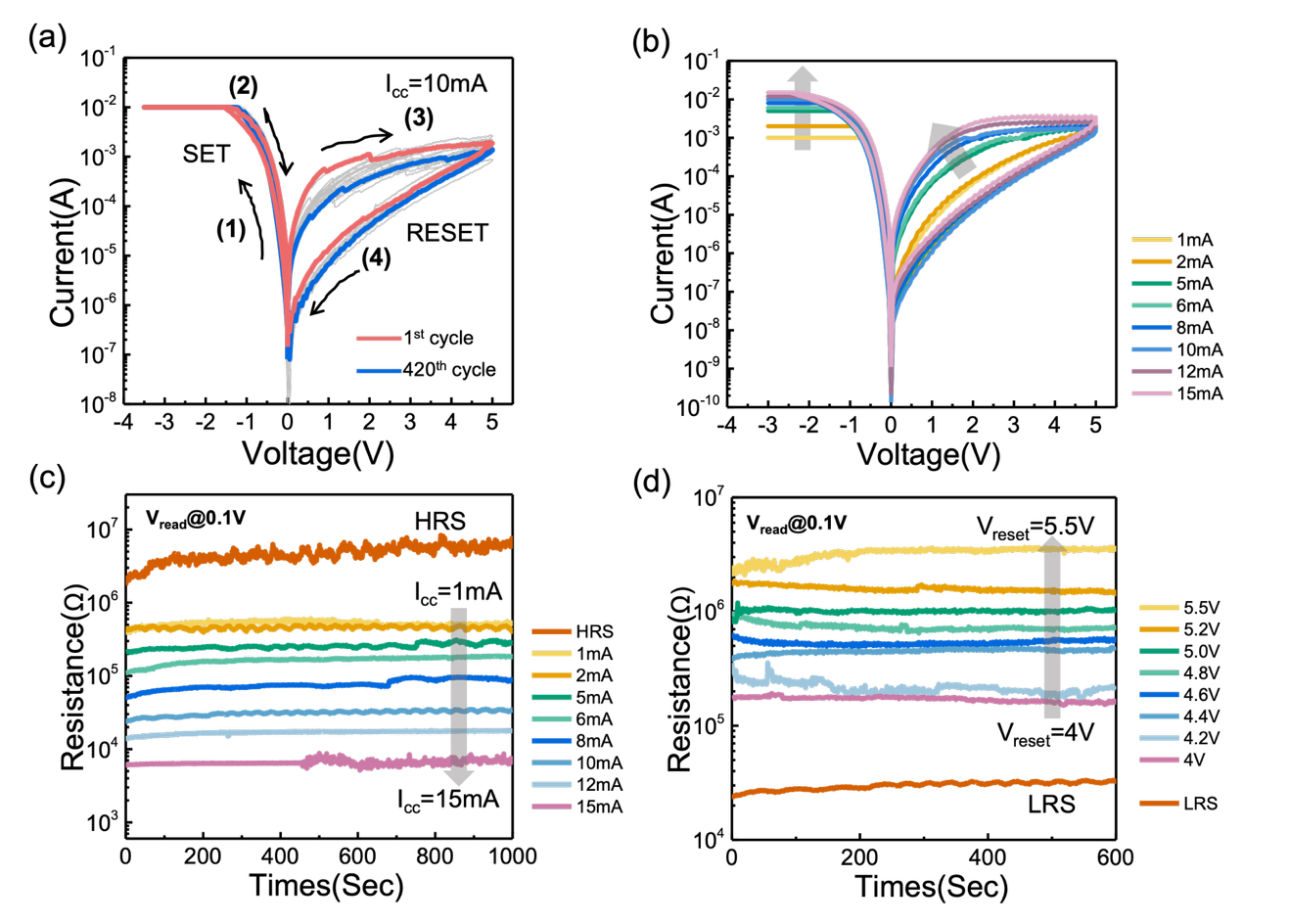


**Figure S4. *I–V* characteristics and multistate switching behavior of the RRAM devices.**

(a) *I–V*characteristics for 420 consecutive DC switching cycles measured on the Au/Ti/Bi_2_O_2_Se/Bi_2_O_5_Se/Nb–STO device with a 10 mA compliance current. (b) *I–V* characteristics of the device with varying SET compliance currents from 1 (yellow line) to 20 mA (pink line). (c) Retention characteristics of multilevel resistive states during a 1000 s reading operation with varying compliance current. A larger compliance current resulted in a smaller LRS. (d) Retention characteristics of multilevel resistive states during a 600 s reading operation with varying RESET voltages from 4 V (yellow line) to 5.5 V (pink line). A larger voltage resulted in a larger HRS.


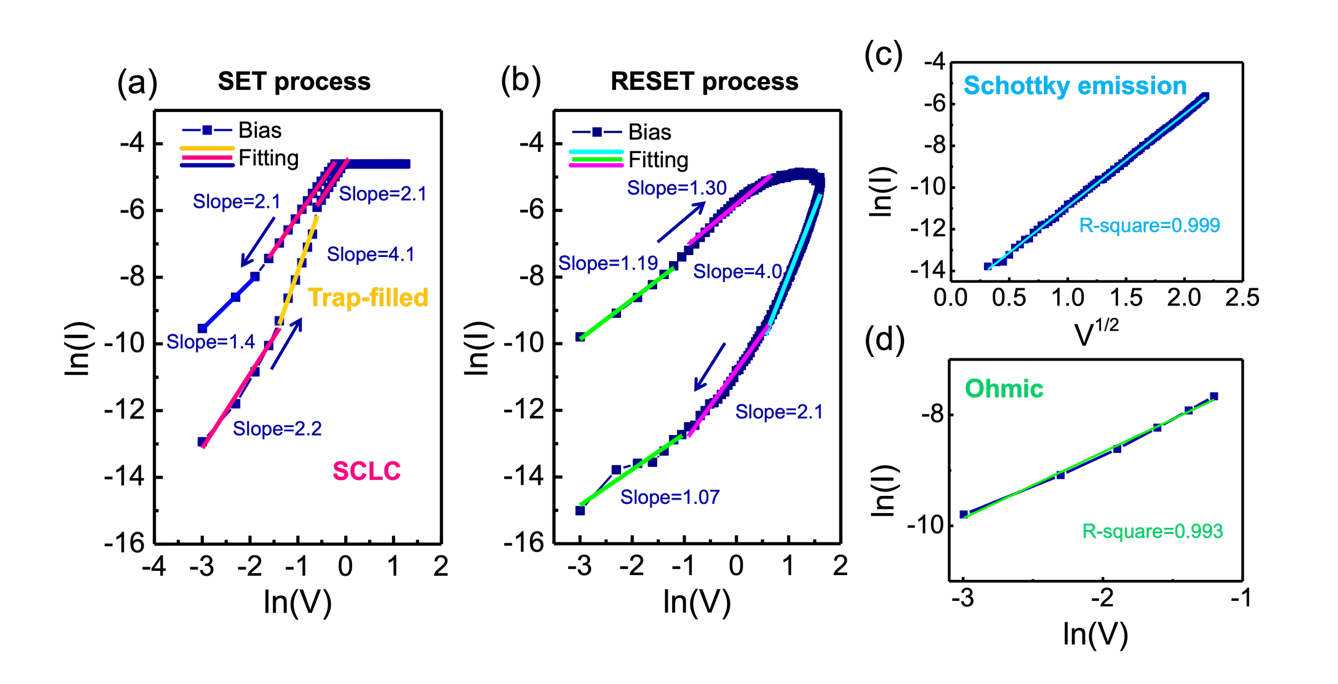


**Figure S5. *I–V* fitting analysis of the resistive switching behavior.**

(a,b) Ln–ln plots of the *I–V* characteristics during the SET and RESET processes.

(c,d) Corresponding fitting plots used to identify the dominant conduction mechanisms.


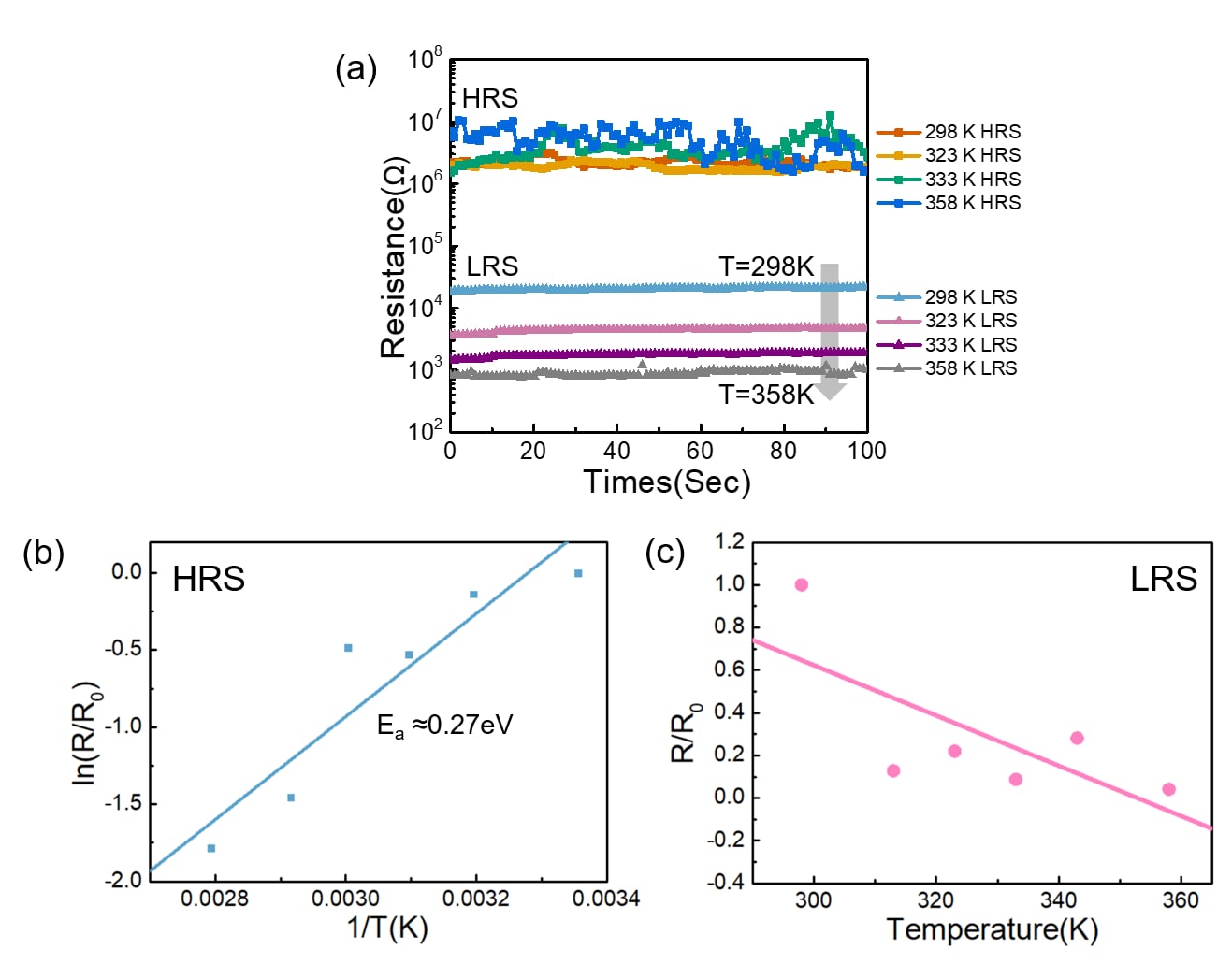


**Figure S6. Temperature impact on retention and switching characteristics of Au/Ti/Bi_2_O_2_Se/Bi_2_O_5_Se/Nb–STO devices.**

(a) Retention performance measured at 298, 323, 333, and 358 K for 100 s, showing a stable on/off ratio over the tested temperature range. (b) Linear fit of 1/T versus ln (R/R_0_) in the HRS. The solid line represents a linear fit based on the Arrhenius equation. (c) Linear fit of T versus R/R_0_ in the LRS. The solid line represents a linear fit to the data.


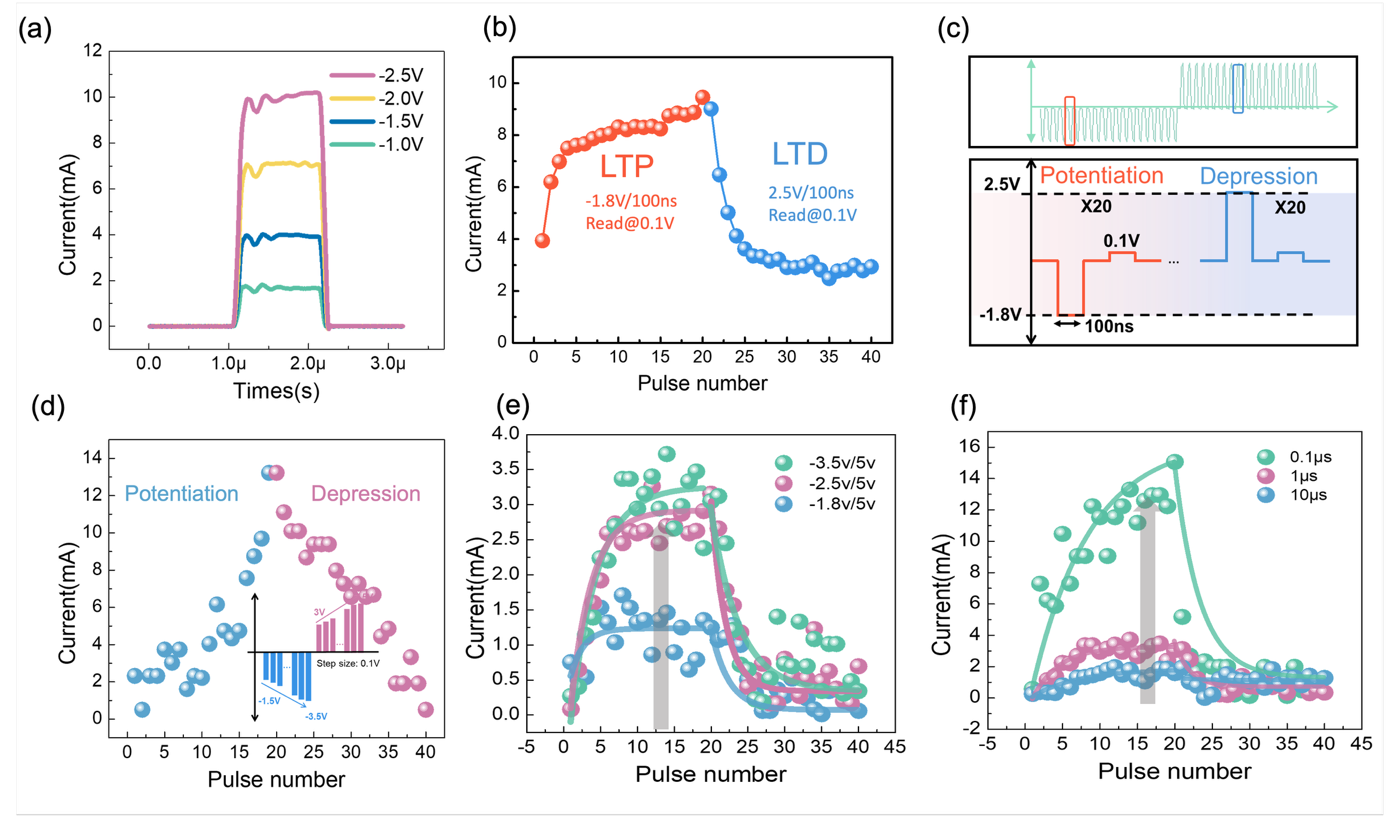


**Figure S7. Synaptic behaviors and current responses of the Au/Ti/Bi_2_O_2_Se/Bi_2_O_5_Se/Nb–STO devices.**

(a) Current responses to applied pulses at various voltages of -1, -1.5, -2, and -2.5 V at a pulse width of 1 μs. (b) Demonstration of the LTP and LTD characteristics of 40 potentiation and depression pulses. (c) Pulse diagram used for potentiation and depression. The amplitude and width of the pulse were -1.8 V/100 ns for the SET process, and 2.5 V/100 ns for the RESET process, with a read voltage of 0.1V. (d) Conductance modulation under pulse programming, where the pulse amplitude was increased from -1.5 to -3.5 V for potentiation and from 3.0 to 5.0 V for depression, both with a step size of 0.1 V and a pulse width of 100 ns. (e) LTP/LTD synaptic behavior obtained under different potentiation voltages of -1.8, -2.5, and -3.5 V. Twenty potentiation pulses followed by 20 depression pulses were applied with a pulse width of 1 μs. (f) LTP/LTD synaptic behavior under different pulse widths of 100 ns, 1 µs and 10 µs. Twenty potentiation pulses followed by 20 depression pulses were applied with a pulse width of 1 μs.


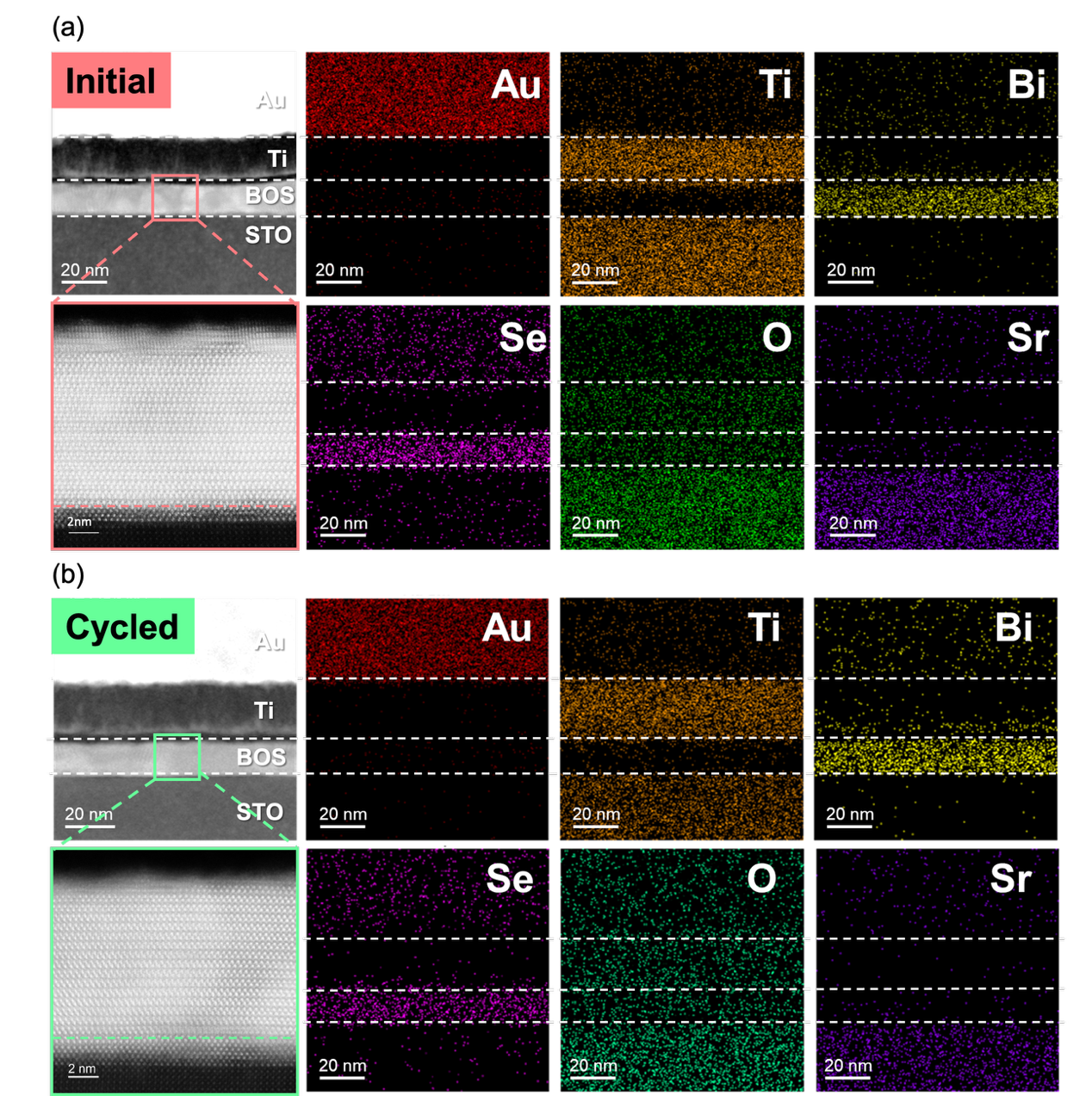


**Figure S8. Cross-sectional STEM–EDS elemental mapping of the RRAM devices before and after electrical measurements.**

(a) In the initial state, the STEM image and enlarged high-resolution view demonstrate sharp and well-defined interfaces, with elemental maps (Au, Ti, Bi, Se, O, and Sr) confirming a uniform layer composition. (b) After electrical cycling, the bilayer structure is preserved, with the bottom layer thickening.


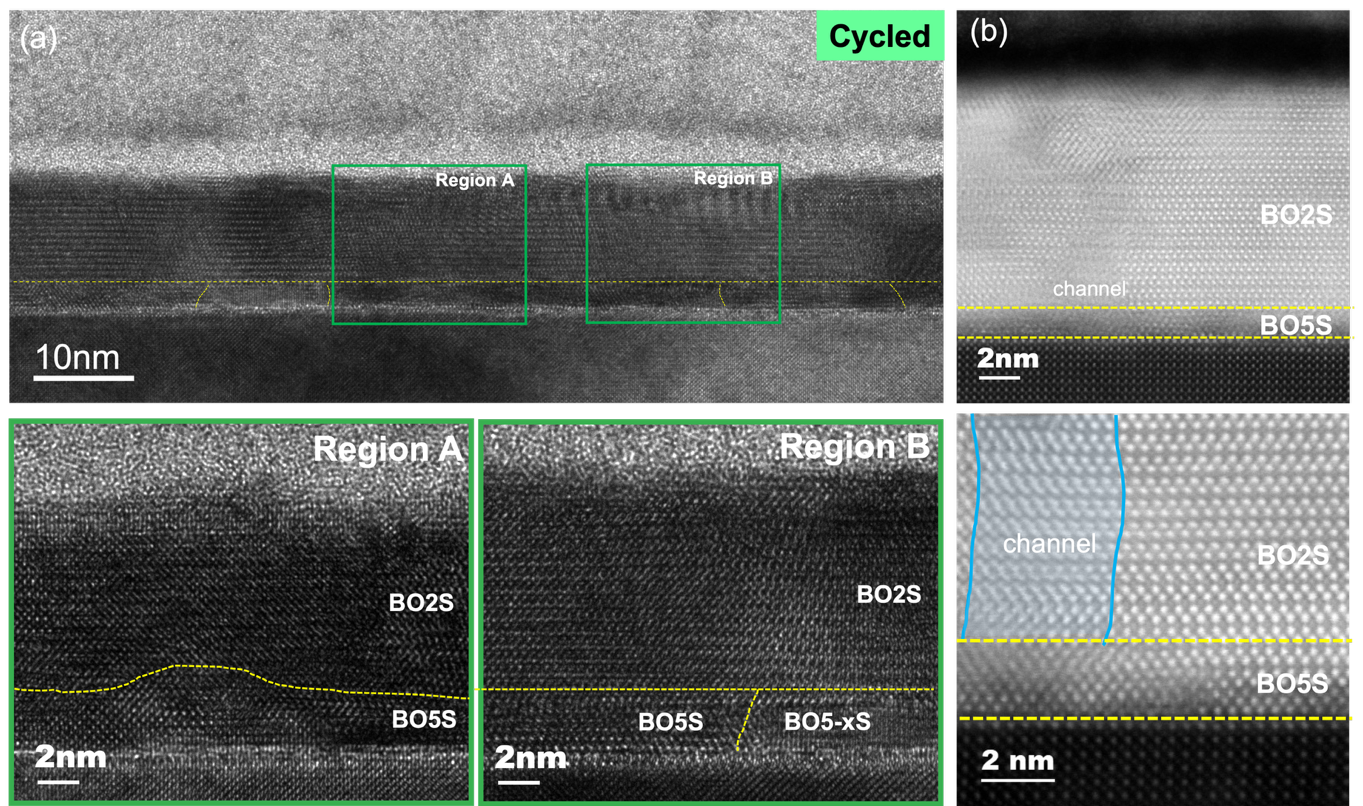


**Figure S9. Wide-field TEM image and STEM images of the cycled device.**

(a) Wide-field TEM image of the cycled device. The enlarged regions A and B indicated in the top image show distinct structural features in the bottom layer after electrical cycling, where both BO5S and BO5-xS regions coexist. (b) The conductive channel in BO2S of the cycled devices.


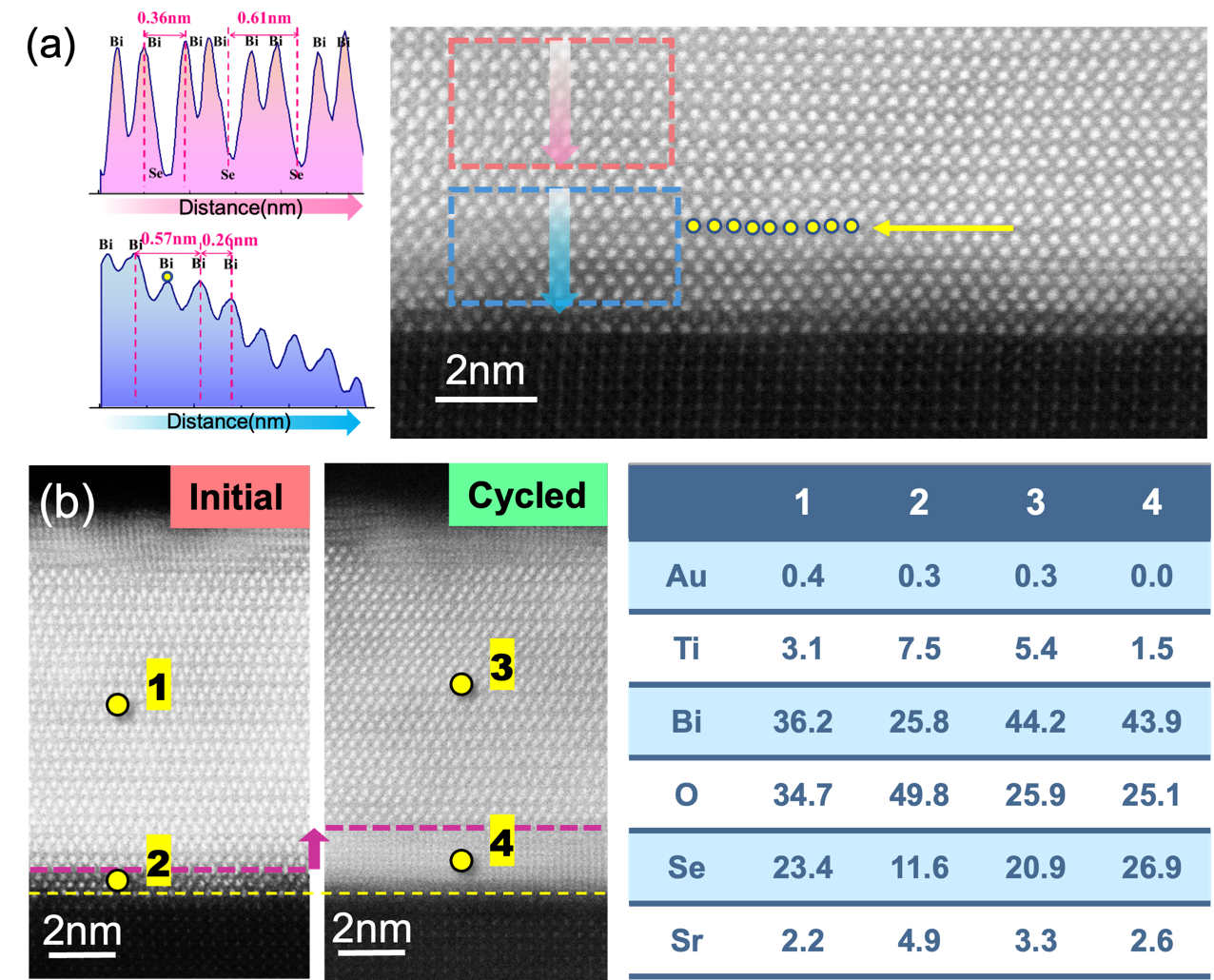


**Figure S10. Microstructural evolution of the Bi_2_O_2_Se/Bi_2_O_5_Se bilayer and EDS point analysis before and after electrical measurements.**

(a) Line profiles showing enlarged interatomic spacings, consistent with the atomic insertion in the lattice. The corresponding STEM image includes boxed regions (red/blue) indicating the line-profile windows. The yellow dotted arrow indicates the insertion region. (b) EDS point analysis (1–4) showing the atomic percentages of Au, Ti, Bi, O, Se, and Sr. In the initial states, the upper layer (point 1) composition was approximately Bi:O:Se ≈ 1.5:1.5:1, corresponding to Bi_2_O_2_Se, and the bottom layer (point 2) ≈ was Bi:O:Se ≈ 2.2:4.3:1, corresponding to Bi_2_O_5_Se. After cycling, the upper layer (point 3) exhibited an O decrease to 25.9 at%. The bottom layer (point 4) exhibited severe O depletion (25.1 at%), corroborating the presence of an oxygen-deficient phase.


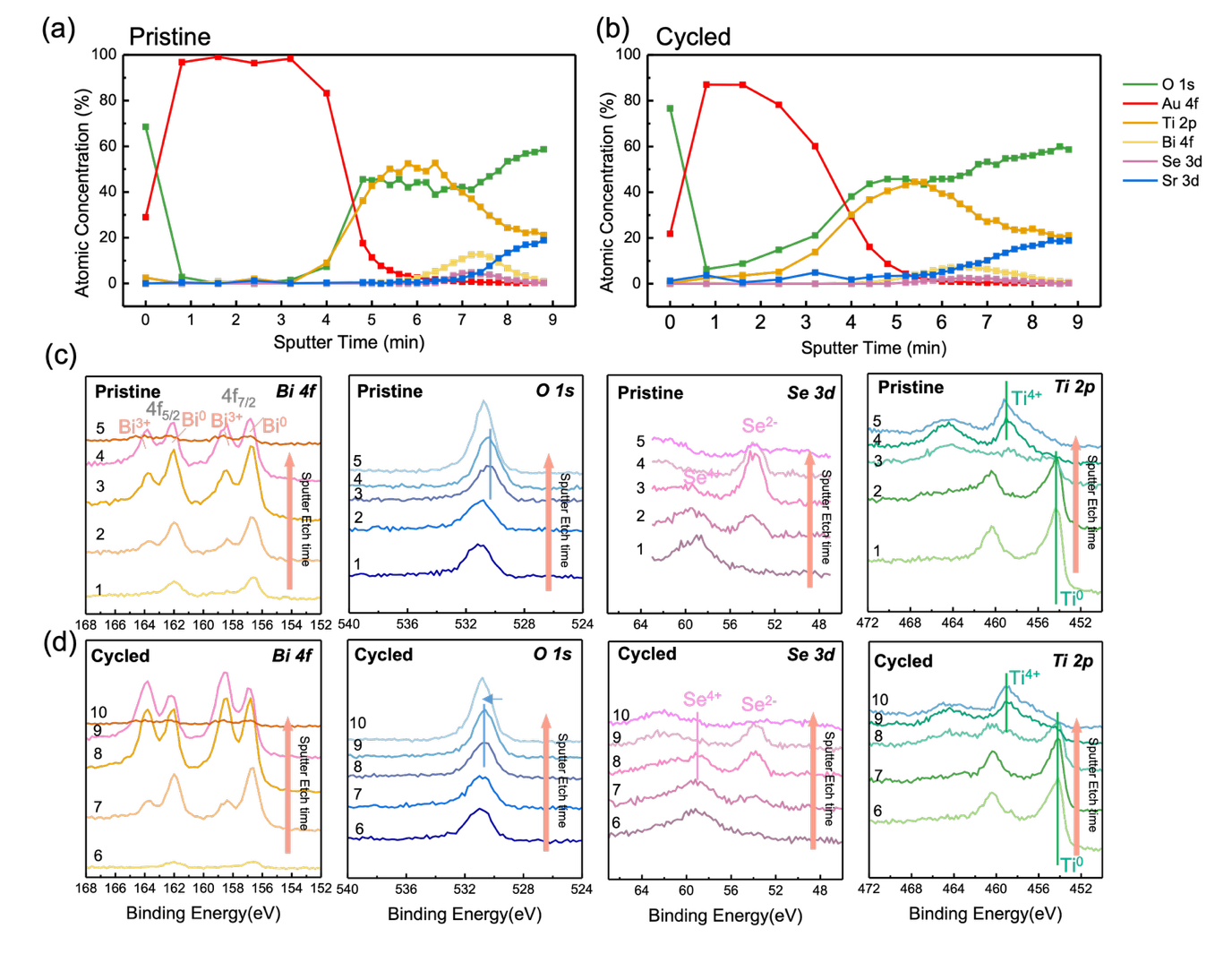


**Figure S11. High-resolution XPS spectra of the pristine and cycled RRAM devices.**

(a,b) XPS depth profiles showing the atomic concentrations of Bi, O, Se, and Ti across the full stack before (a) and after (b) electrical treatments. (c,d) Depth-resolved XPS spectra of Bi 4f, O 1s, Se 3d, and Ti 2p acquired from the top electrode toward the substrate before (c) and after (d) electrical cycling.


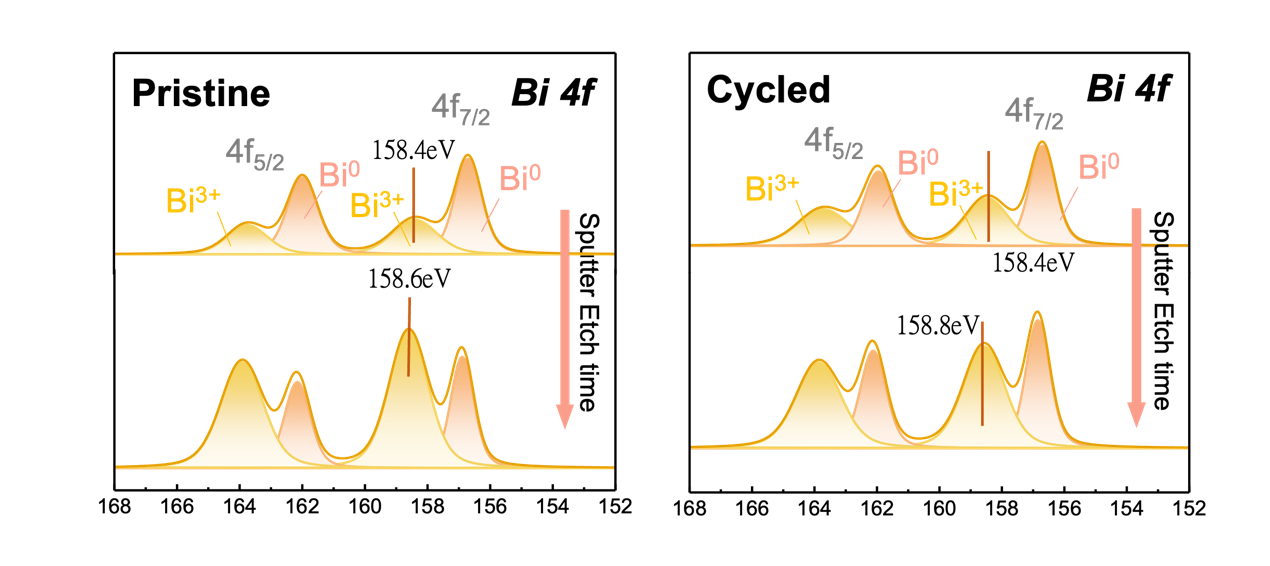


**Figure S12. XPS spectra of Bi 4f for the pristine and cycled devices.**

The upper and bottom spectra correspond to the Bi_2_O_2_Se and Bi_2_O_5_Se layers, respectively.


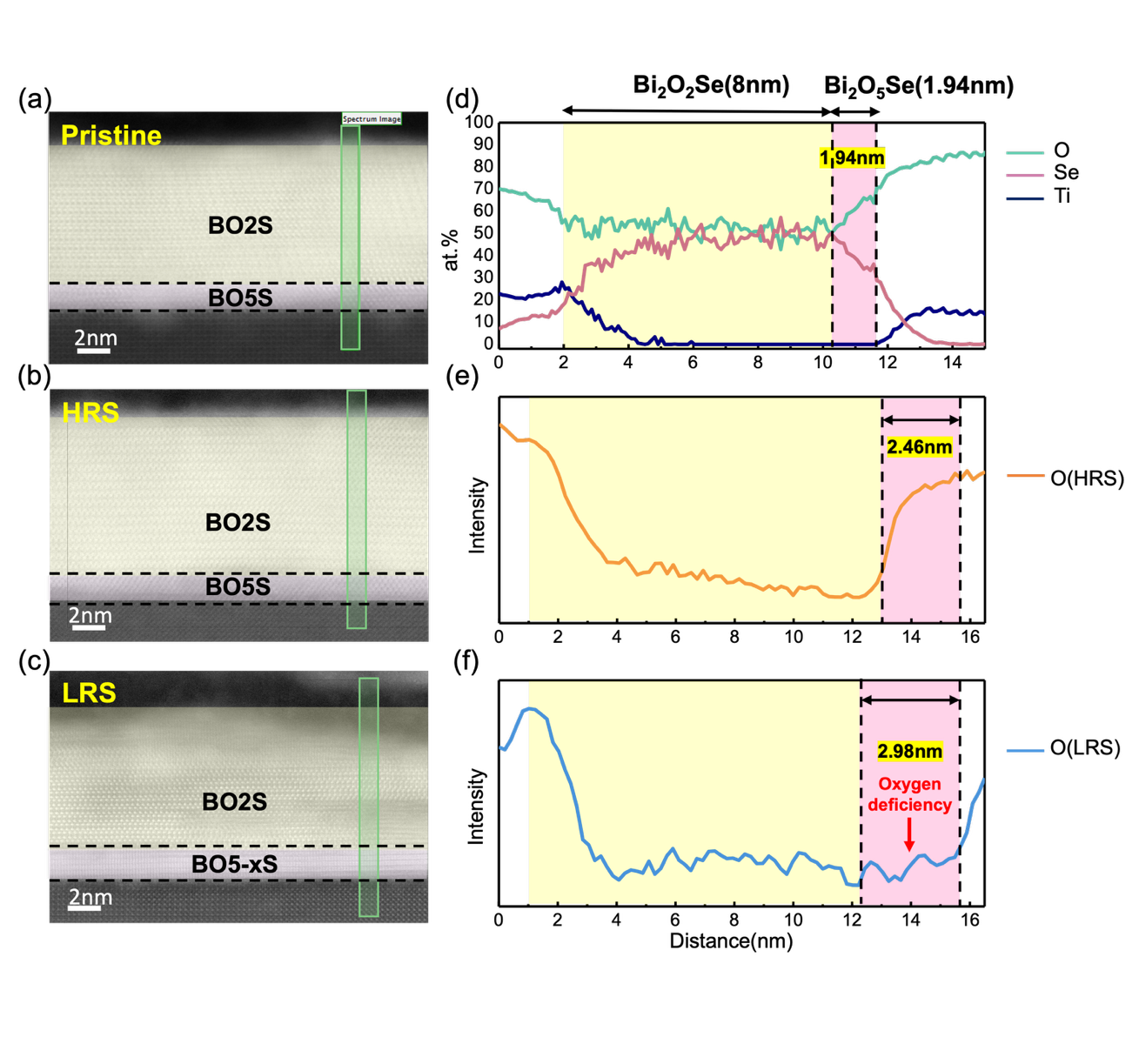


**Figure S13. STEM–EELS analysis of the interfacial evolution in the RRAM device.**

(a–c) Cross-sectional STEM images of the device in the initial, cycled-HRS, and cycled-LRS conditions, respectively. The colored overlays indicate the Bi_2_O_2_Se (yellow) and Bi_2_O_5_Se (pink) layers. The green rectangle indicates the area used for EELS line-scan measurements. (d–f) Corresponding EELS line profiles showing the elemental distributions across the Bi_2_O_2_Se/Bi_2_O_5_Se interface in different resistance states.


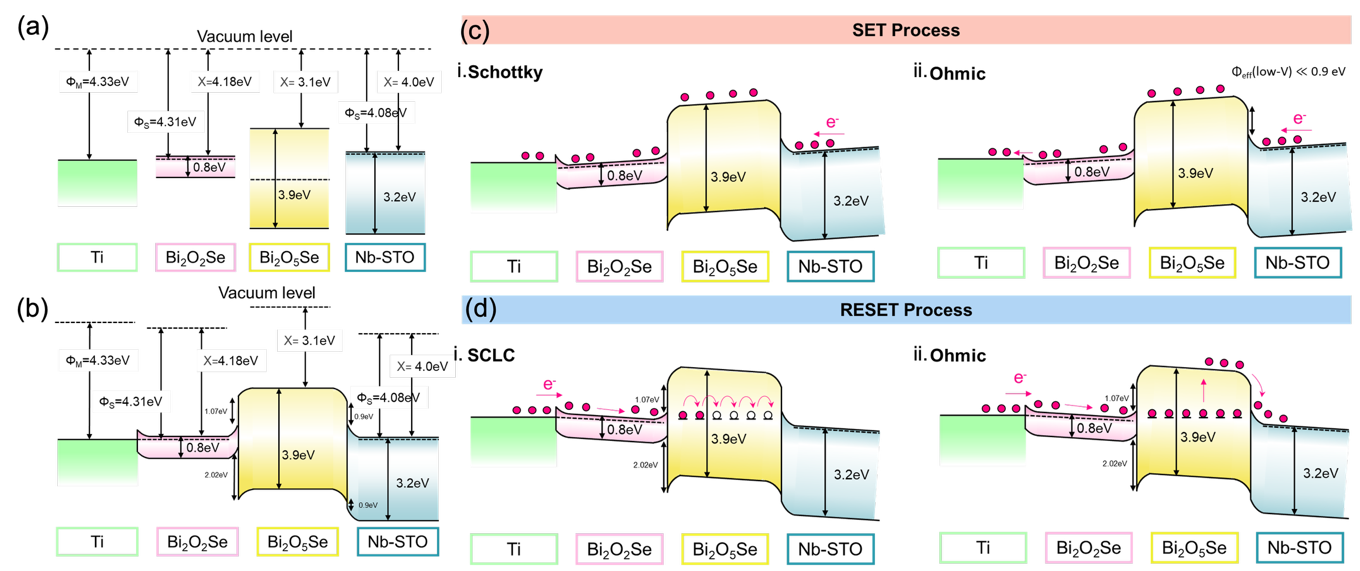


**Figure S14. Energy-band diagrams and resistive switching mechanism.**

 Work function and energy gap of each layer (a) before and (b) after contact. The diagram illustrates the movement of electrons during the (c) SET and (d) RESET processes.


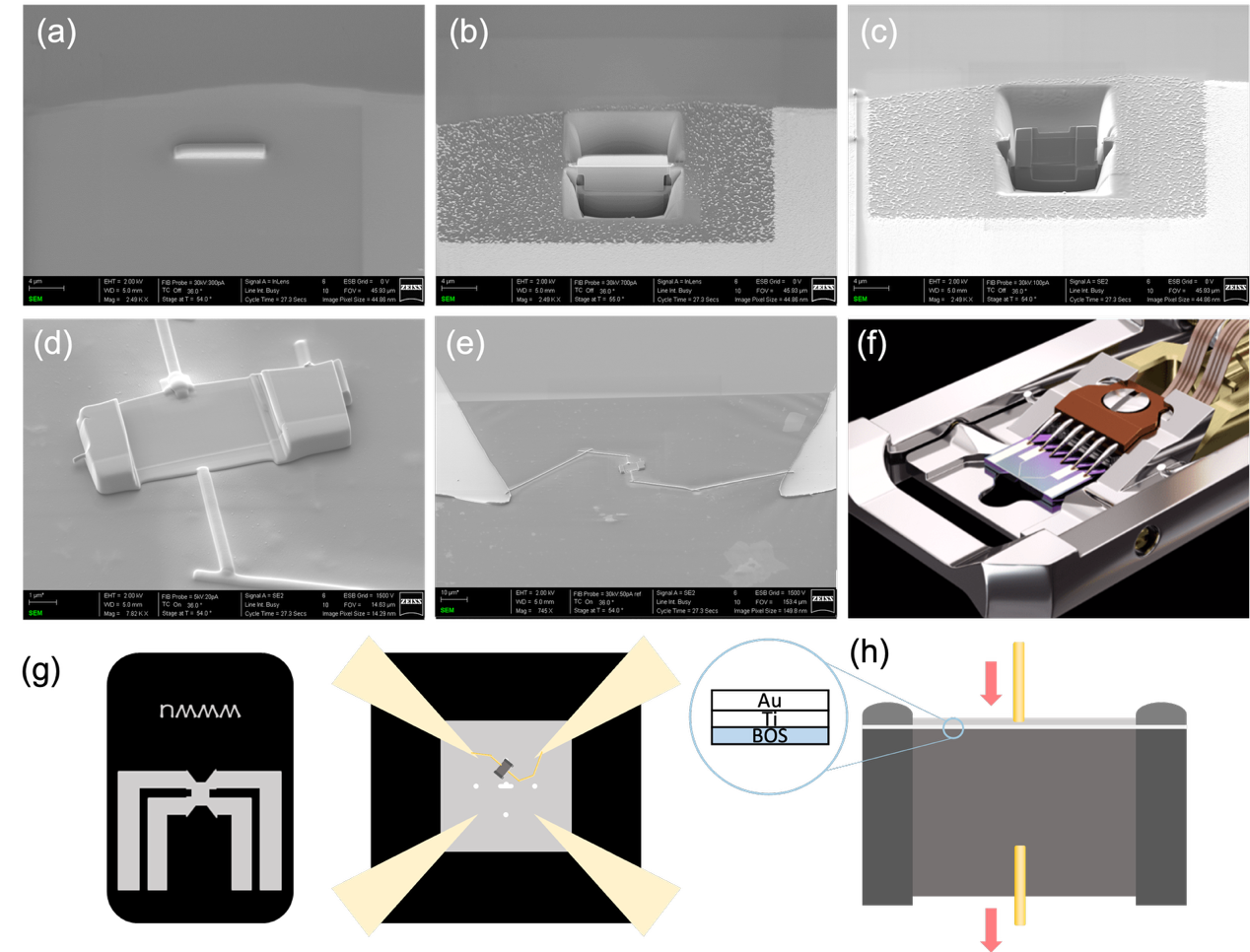


**Figure S15. Experimental setup for *in situ* TEM analysis using FIB system and E-chip holder.** (a–e) SEM images of the sample preparation process. (f) The E-chip is mounted on the tip of the Fusion Select holder, which contains electrical probes for bias application. (g) Schematic of the E-chip. The bias is applied through two selected terminals. (h) The direction of the electron flow is perpendicular to the MIM structure.

**Table S1.** **Comparison of the Bi_2_O_2_Se/Bi_2_O_5_Se heterostructure RRAM device with previous memristors.**

In this table, we compared the Au/Ti/Bi_2_O_2_Se/Bi_2_O_5_Se/Nb-STO RRAM device with previously reported Bi_2_O_2_Se-, Bi_2_O_5_Se-, and related bilayer-based memristive systems. The device architecture, ON/OFF ratio, switching speed, endurance under DC and pulsed operations, and demonstrated synaptic functions were evaluated.

| **Architectures** | **ON/OFF ratio** | **Switching speed**  **SET/RESET** | **Endurance**  **(DC cycles)** | **Endurance**  **(Pulse cycles)** | **Synaptic functions** | **Reference** |
| --- | --- | --- | --- | --- | --- | --- |
| (Al/Cu)/Bi_2_O_2_Se/Pd | 10^1^ | - | 90 | >3.5 × 10^3^ | LTP/LTD | Ref.^[13]^ |
| Ag/Bi_2_SeO_5_/Au | 10^8^ | 40ns/- | 50 | - | LTP/LTD, STP | Ref.^[14]^ |
| Au/Bi_2_SeO_5_/Au | 10^6^ | 160ns/110ns | 1000 | - | Memristive switching | Ref.^[15]^ |
| Ag/Bi_2_O_2_Se/Au | 10^3^ | 5ns/41ns | 75 | - | LTP/LTD,PPF,STDP | Ref.^[16]^ |
| (Cr/Au)/Bi_2_O_2_Se/Oxide layer/(Cr/Au) | 10^1^ | - | 20 | 65 | Memristive switching | Ref.^[17]^ |
| Cr/β-Bi_2_SeO_5_/Bi_2_O_2_Se/Au | 10^5^ | 20ns/- | 20 | - | Memristive switching | Ref.^[18]^ |
| Au/Ti/Bi_2_O_2_Se/Bi_2_O_5_Se/Nb-STO | 10^3^ | 20ns/100ns | >600 | >3 × 10^6^ | LTP/LTD,PPF,STDP | This work |

**References**

[1] T. Li, T. Tu, Y. Sun, H. Fu, J. Yu, L. Xing, Z. Wang, H. Wang, R. Jia, J. Wu, *Nature Electronics* **2020**, 3, 473.

[2] J. Wu, C. Tan, Z. Tan, Y. Liu, J. Yin, W. Dang, M. Wang, H. Peng, *Nano Letters* **2017**, 17, 3021.

[3] Y. Y. Hsieh, Y. C. Chuang, H. Y. Tuan, *Advanced Functional Materials* **2024**, 34, 2406975.

[4] Y. Cao, Y. Qian, L. Chen, T. Song, Y. Pei, X. Wang, X. Wu, Y. Dong, F. Liu, B. Long, *Journal of Energy Storage* **2025**, 125, 116986.

[5] E. W. Lim, R. Ismail, *Electronics* **2015**, 4, 586.

[6] P. Zhang, Y. S. Ang, A. L. Garner, Á. Valfells, J. Luginsland, L. Ang, *Journal of Applied Physics* **2021**, 129.

[7] T. Kim, T. Vogel, E. Piros, D. Nasiou, N. Kaiser, P. Schreyer, R. Winkler, A. Zintler, A. Arzumanov, S. Petzold, *Applied Physics Letters* **2023**, 122.

[8] C.-Y. Lin, T.-C. Chang, C.-H. Pan, M.-C. Chen, Y.-L. Xu, Y.-F. Tan, P.-Y. Wu, C.-K. Chen, W.-C. Huang, Y.-H. Lin, *Journal of Alloys and Compounds* **2020**, 817, 150566.

[9] L. Wang, Q. Zhang, L. Chang, L. You, X. He, K. Jin, L. Gu, H. Guo, C. Ge, Y. Feng, *Advanced Electronic Materials* **2017**, 3, 1700321.

[10] A. Sharma, N. Sharotri, P. Kandwal, R. K. Sharma, D. Sud, R. Rai, A. Hnydiuk-Stefan, *Scientific Reports* **2024**, 14, 13677.

[11] C. Chen, M. Wang, J. Wu, H. Fu, H. Yang, Z. Tian, T. Tu, H. Peng, Y. Sun, X. Xu, *Science Advances* **2018**, 4, eaat8355.

[12] L. Zhao, Z. Lu, F. Zhang, G. Tian, X. Song, Z. Li, K. Huang, Z. Zhang, M. Qin, SujuanWu, *Scientific reports* **2015**, 5, 9680.

[13] D. Verma, T.-C. Chen, B. Liu, C.-S. Lai, *Heliyon* **2023**, 9.

[14] F. Yang, Y. Xiong, Z. Chen, S. Wang, Y. Wang, Z. Qu, W. Zhao, J. Li, K. Yin, Z. Ni, *Advanced Functional Materials* **2025**, e14338.

[15] T. Guo, Z. Pan, Y. Shen, J. Yang, C. Chen, Y. Xiong, X. Chen, Y. Song, N. Huo, R. Xu, *Nano Letters* **2025**, 25, 8258.

[16] Z. Dong, Q. Hua, J. Xi, Y. Shi, T. Huang, X. Dai, J. Niu, B. Wang, Z. L. Wang, W. Hu, *Nano Letters* **2023**, 23, 3842.

[17] Y. Xia, J. Wang, R. Chen, H. Wang, H. Xu, C. Jiang, W. Li, X. Xiao, *Advanced Electronic Materials* **2022**, 8, 2200126.

[18] Y. Zhao, Z. Lou, J. Hu, Z. Li, L. Xu, Z. Chen, Z. Xu, T. Wang, M. Wu, H. Ying, *Advanced Materials* **2024**, 36, 2406608.
